# CircRNA *GRAMD4* induces *NBR1* expression to promote autophagy and immune escape in renal cell carcinoma

**DOI:** 10.1080/15548627.2025.2503560

**Published:** 2025-05-15

**Authors:** Mi Zhou, Minyu Chen, Zhousan Zheng, Qihao Li, Lican Liao, Yunfei Wang, Yi Xu, Guannan Shu, Junhang Luo, Taowei Yang, Jiaxing Zhang

**Affiliations:** a Department of Oncology, The First Affiliated Hospital of Sun Yat-sen University, Guangzhou, Guangdong, PR China; b Department of Urology, The First Affiliated Hospital of Sun Yat-sen University, Guangzhou, Guangdong, PR China; c Department of Breast and Thyroid Surgery, Guangzhou Women and Children’s Medical Center, Guangzhou Medical University, Guangzhou Provincial Clinical Research Center for Child Health, Guangdong, PR China; d Department of Urology, Guangzhou Women and Children’s Medical Center, Guangzhou Medical University, Guangzhou, Guangdong Provincial Clinical Research Center for Child Health, Guangdong, PR China

**Keywords:** Autophagy, circRNA, immune evasion, renal cell carcinoma, CD8^+^ T cells

## Abstract

The tumor microenvironment (TME) in renal cell carcinoma (RCC) frequently exhibits significant immune cell infiltration. However, tumor cells often manage to evade immune surveillance. This study revealed the mechanism by which circular RNA *circGRAMD4* regulates *NBR1*. *CircGRAMD4* is markedly elevated in RCC, and its high levels are correlated with a poor prognosis. Notably, the absence of *circGRAMD4* has been demonstrated to result in a significant inhibition of renal cancer cell growth. This inhibition has been attributed to an enhanced anti-tumor immunity mediated by CD8^+^ T cells. Mechanistically, *circGRAMD4* interacts with the RBM4 protein, stabilizing the autophagic cargo receptor *NBR1* mRNA. This interaction promotes *NBR1* expression, which in turn leads to the degradation of MHC-I molecules through macroautophagy/autophagy pathways. Consequently, this process affects renal cancer cell antigen presentation, induces CD8^+^ T cell dysfunction, and contributes to tumor immune escape. Moreover, by inhibiting *circGRAMD4* and using immune checkpoint blockers (ICB), the immunosuppressive TME is altered to prevent tumor immune evasion, ultimately increasing the effectiveness of ICB treatment. The discovery highlights the significant impact of *circGRAMD4* on RCC immune escape and proposes that blocking *circGRAMD4* could serve as a promising immunotherapy strategy when combined with ICB to enhance patient outcomes.

## Introduction

Renal cell carcinoma (RCC) is a prevalent malignant tumor affecting the urinary system worldwide [1]. Despite advancements in systemic therapies, nephrectomy remains the primary treatment option for RCC. Notably, significant progress has been made in treating metastatic RCC, including approaches that combine anti-tumor angiogenesis and immunotherapy [2–4]. RCC often exhibits significant immune infiltration within the TME [5,6]. Immune checkpoint blockers (ICBs) have emerged as crucial components of treatment strategies, contributing to improved survival for metastatic RCC patients [7–9]. Nevertheless, the emergence of secondary or acquired ICB resistance hinders long-term treatment benefits for patients [10]. Hence, there is a pressing need to pinpoint possible causative factors and novel treatment objectives for immune avoidance in RCC, with the aim of enhancing the responsiveness of RCC patients to ICB therapy.

CD8^+^ T cells play a vital role in the anti-tumor immune reaction. However, in the RCC microenvironment, these cells frequently experience dysfunction and exhaustion [11,12]. Notably, unlike most solid tumors, high infiltration of CD8^+^ T cells in RCC is linked to poorer survival outcomes in patients [13]. Research findings suggest that detecting evidence of CD8^+^ T cells depletion in tumor samples before and during early treatment can serve as a predictor for the favorable clinical effects of ICB therapy [14].

Circular RNAs (circRNAs) are a distinct group of natural ribonucleic acids. Unlike linear RNAs, circRNAs form a continuous loop, rendering them resistant to exonuclease-mediated degradation [15]. CircRNAs are widely distributed across eukaryotic cells and have been identified in various species, including humans [16]. In terms of their functional role, circRNAs are involved in the regulation of gene expression. This is achieved through their ability to act as sponges, interact with RNA-binding proteins and even code for proteins, suggesting that they have significant roles in transcriptional and post-transcriptional modifications [17]. Their inherent stability and detection in bodily fluids position them as promising candidates for both biomarkers and therapeutic targets [18,19].

Autophagy plays a vital role in maintaining cell homeostasis and responding to stress conditions [20,21]. While autophagy was previously regarded as a nonselective, large-scale degradation process, recent evidence confirms the existence of multiple selective autophagy pathways [22]. In selective autophagy, specific substrates are recognized by various cargo receptors, including SQSTM1/p62, BNIP3L/NIX, NBR1, CALCOCO2/NDP52, OPTN, and others [23]. Notably, recent studies have demonstrated that autophagy contributes to immune evasion in pancreatic cancer by selectively degrading MHC-I (major histocompatibility complex, class I) molecules [24,25]. However, whether autophagy is involved in immune escape mechanisms in RCC remains unclear.

In our study, we discovered that *circGRAMD4* correlates with patient prognosis and the infiltration of CD8^+^ T cells in the TME of RCC. Mechanistically, *circGRAMD4* forms a ternary complex with RBM4 protein and *NBR1* mRNA, leading to increased stability of *NBR1* mRNA and subsequent upregulation of *NBR1*, which mediates the autophagy of MHC-I, resulting in reduced MHC-I levels within RCC cells. This process affects tumor antigen presentation and CD8^+^ T cells function, ultimately leading to CD8^+^ T cells depletion and promoting immune escape in RCC. Additionally, the simultaneous blocking of *circGRAMD4* and immune checkpoint demonstrates a stronger anti-cancer impact in RCC preclinical patient-derived xenograft (PDX) models when compared to using only one treatment. The current research demonstrates the significant impact of *circGRAMD4* on enhancing immune evasion, suggesting a possible enhancement for ICB therapy in RCC.

## Results

### Discovery and characterization of a novel circular RNA, circGRAMD4, which exhibits a positive correlation with CD8^+^ T cells infiltration in RCC

**Figure 1.**
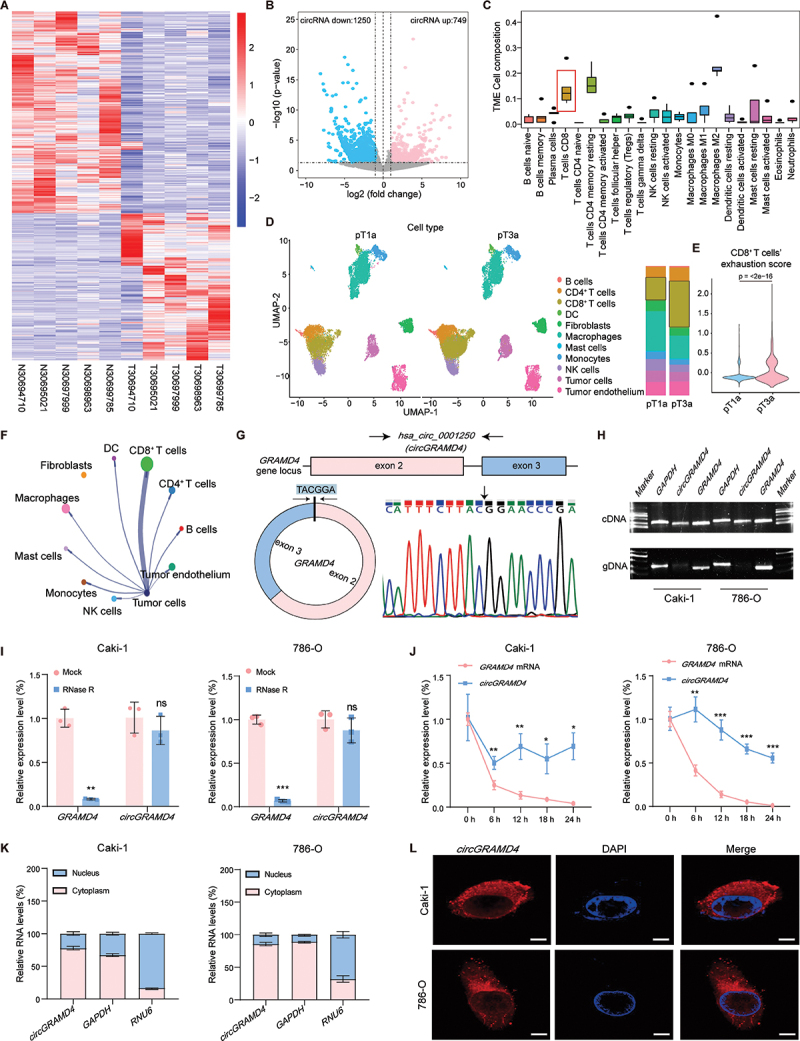
Discovery and characterization of a novel circular RNA, *circGRAMD4*, which exhibits a positive correlation with CD8^+^ T cells infiltration in RCC. (A) The heatmap shows the differentially expressed circRNAs in 5 pairs of RCC tissues. (B) Volcano plot of differentially expressed circRNAs in 5 pairs of human RCC samples. (C) TME cell composition of 5 tumor tissues. (D) UMAP plot and bar plot displaying the identified cell subset clusters and their proportion in the indicated groups. (E) Violin plot of CD8^+^ T cell exhaustion scores for each group. (F) Communication network diagram between tumor cells and other cells. (G) Schematic illustration of *circGRAMD4*. The junction site was verified by Sanger sequencing and is indicated by a black arrow. (H) PCR products with divergent or convergent primers in cDNA and gDNA of caki-1 and 786-O cells, evaluated by agarose gel electrophoresis. (I) qRT-PCR analysis of *circGRAMD4* and linear *GRAMD4* in RCC cells treated or not treated with RNase R. (J) Relative abundance of *circGRAMD4* and linear *GRAMD4* in caki-1 and 786-O cells treated with actinomycin D at the indicated time points. (K) Subcellular location of *circGRAMD4* in caki-1 and 786-O cells detected by nuclear and cytoplasmic separation assay. (L) FISH assay showed that *circGRAMD4* is mainly localized in the cytoplasm. Scale bar: 5 μm. Data are shown as mean± SD; *p < 0.05, **p < 0.01, ***p < 0.001.

In order to identify dysregulated circRNAs in RCC, microarray data were initially examined, which included the expression profiles of circRNAs in five paired RCC tissues (Figure 1A). Applying the pre-established criteria (|log2 fold change| > 1, and *p* < 0.05), we identified 1999 dysregulated circRNAs (Data S1). Among them, 749 circRNAs were upregulated, while 1250 circRNAs were downregulated in RCC (Figure 1B). Additionally, we performed transcriptome sequencing on the same 5 paired RCC tissues and analyzed the proportion of immune cells using the CIBERSORT algorithm (Figure S1A). Upon independent analysis of the immune cell components within the TME of the five tumor tissues, it was observed that there was a high proportion of CD8^+^ T cells (Figure 1C). Additionally, analysis of transcriptome sequencing data from 542 RCC cancer tissues in the TCGA database revealed substantial infiltration of CD8^+^ T cells in RCC tissues (Figure S1B). Similarly, we analyzed the scRNA-seq data of RCC tumor samples from the GSE242299 dataset in the GEO database. After quality control and batch calibration, a total of 31,117 cells were obtained for subsequent analysis (Figure S1C,D). We found that tumor tissues from RCC patients with pT3a had a higher proportion of CD8^+^ T-cell infiltration (Figure 1D) and exhaustion (Figures 1E, S1E) than that from RCC patients with pT1a. Cellchat analysis showed that in pT3a phase, tumor cells mainly sent signals to CD8^+^ T cells (Figure 1F). The heatmap of incoming signaling patterns and receptor-ligand analysis showed that the tumor cells and CD8^+^ T cells intercellular communication was mainly transmitted through MHC-I in pT3a phase (Figure S1F-G). To further identify candidate circRNAs involved in immune escape, we subjected circRNAs to weighted gene co-expression network analysis (WGCNA), resulting in the identification of 45 circRNA modules (power = 12, Figure S2A-B). The analysis of the module-trait relationship indicated a positive correlation between the expression of circRNAs within the orangered4 module and CD8^+^ T cells (Figure S2C, Data S2). Furthermore, based on intramodular analysis, we identified 42 hub circRNAs from the orangered4 module (gene significance > 0.91, module membership correlation = 0.84, q-weighted value < 0.01) (Figure S2D).

Based on the results of WGCNA analysis and differential analysis, five circRNAs (*hsa_circ_0001250*, *hsa_circ_0000724*, *hsa_circ_0003513*, *hsa_circ_000_2193*, and *hsa_circ_0003861*) were identified as exhibiting a positive correlation with CD8^+^ T cells infiltration and exhibiting high expression levels in the tumor tissues (Figure S2E). To further identify candidate circRNA, we conducted differential analysis on the GEO dataset (GSE100186) and found that *has_circ_0001250* was also highly expressed in RCC tissues (log2 fold change = 1.297, *p* = 0.044). Therefore, we take *hsa_circ_001250* as our further research object (Figure S2E). *Hsa_circ_0001250*, also known as *circGRAMD4*, originates from the *GRAMD4* (GRAM domain containing 4) gene located on chromosome 22 and is formed through reverse splicing of exons 2 and 3 (332 bp). To validate the back-splicing junctions of *circGRAMD4*, we employed Sanger sequencing (Figure 1G). Additionally, the online database RNAfold WebServer was used to determine the secondary structure of *circGRAMD4* (http://rna.tbi.univie.ac.at/cgi-bin/RNAWebSuite/RNAfold.cgi) (Figure S2F). Two RCC cell lines (786-O, Caki-1) exhibited increased *circGRAMD4* expression compared to the typical kidney epithelial cell line HK2, while A498 displayed decreased *circGRAMD4* expression (Figure S2G). PCR analysis verified that divergent primers were able to amplify *circGRAMD4* from cDNA, but not from genomic DNA (Figure 1H). In addition, *circGRAMD4* demonstrated greater resistance to RNase R treatment, whereas *GRAMD4* mRNA was notably degraded by the same treatment (Figure 1I). *CircGRAMD4* also exhibited a longer half-life compared to *GRAMD4* mRNA, which was validated through actinomycin D treatment (Figure 1J). Subcellular fractionation and fluorescence in situ hybridization (FISH) assays suggested that *circGRAMD4* predominantly localized to the cytoplasm in RCC cells (Figure 1K,L). We also discovered that patients with elevated levels of *circGRAMD4* in the tumor tissue of RCC exhibited a higher proportion of tumor infiltration by CD8^+^ T cells (Figure S2H). Subsequently, we used qRT-PCR to examine *circGRAMD4* levels in 102 T1–3 RCC cases that underwent surgery. Notably, *circGRAMD4* expression significantly increased in T2 and T3 stages compared to patients in the T1 stage (Figure S2I). Importantly, high *circGRAMD4* expression was positively associated with dismal OS and PFS (Figure S2J, K). The aforementioned findings indicate that elevated *circGRAMD4* is positively correlated with the proportion of CD8^+^ T cells in RCC, but also with poor prognosis in RCC patients.

### *CircGRAMD4* suppresses the CD8^+^ T cell response against RCC in vitro and in vivo

**Figure 2.**
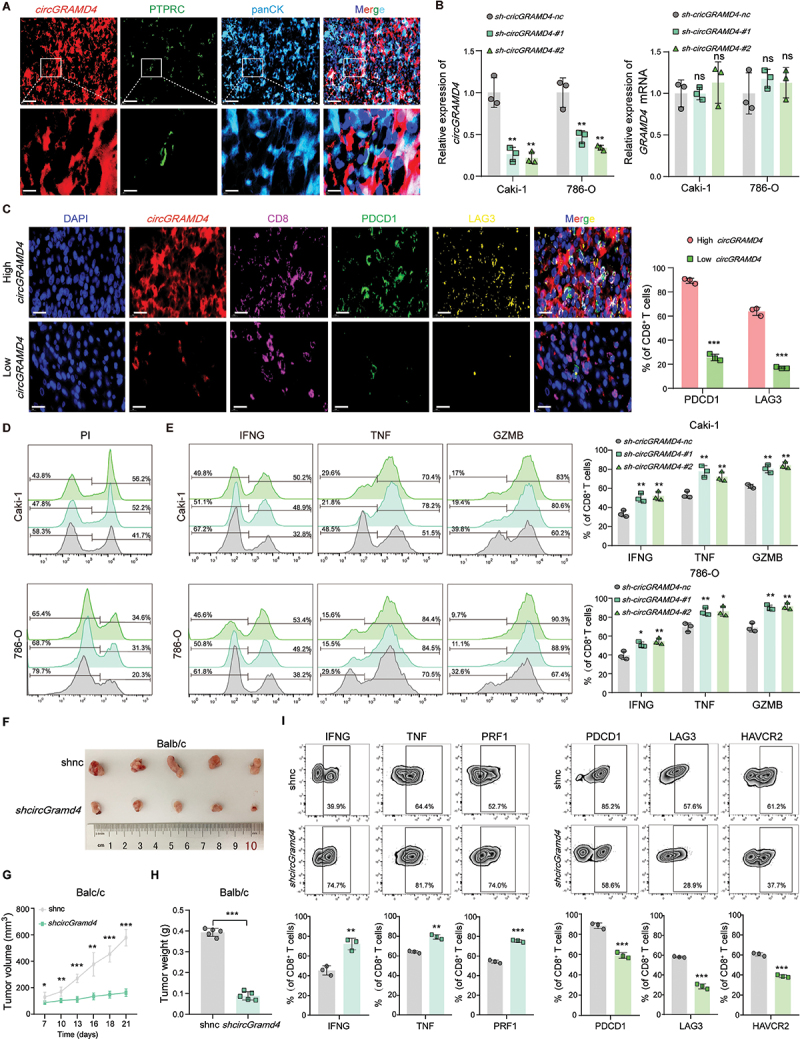
*CircGRAMD4* suppresses the CD8^+^ T cell response against RCC in vitro and in vivo. (A) The results of FISH-IF staining using tumor tissue slices from RCC patients showed that *circGRAMD4* was mainly expressed in RCC cells rather than immune cells. (B) The knockdown efficiency was measured by qRT-PCR in caki-1 and 786-O cell lines. (C) The mfIHC analysis detected CD8^+^ T cells depletion related indicators in renal cancer tissue sections with high and low expression of *circGRAMD4*, respectively. (D) Representative flow cytometry images of death rate (PI^+^) of primary kidney tumor cells with or without *circGRAMD4* knockdown cocultured with primary kidney tumor-specific CD8^+^ T cells. (E) Representative images and statistical quantification of the FACS analysis of the percentage of IFNG^+^, TNF^+^, and GZMB^+^ CD8^+^ T cells cocultured with primary kidney tumor cells with or without *circGRAMD4* knockdown. (F) Images of the collected subcutaneous xenograft tumors from BALB/c mice. (G) Record the tumor volume of tumor bearing BALB/c mice every 3 days. (H) Eventual weights of subcutaneous xenograft tumors. (I) Flow cytometry analysis of killing factor (IFNG, TNF, and PRF1) and immunosuppressive molecules (PDCD1, LAG3, and HAVCR2) in CD8^+^ T cells isolated from Renca tumors in BALB/c mice. Data are shown as mean± SD; *p < 0.05, **p < 0.01, ***p < 0.001.

We observed that *circGRAMD4* exhibited preferential expression in RCC cells compared to immune cells in RCC patients (Figures 2A, S3A). To manipulate *circGRAMD4* expression, we constructed shRNAs targeting the back-splicing junction. The shRNAs effectively reduced the expression of *circGRAMD4* in two RCC cell lines (Caki-1 and 786-O) that had the highest levels of *circGRAMD4*, while leaving the expression of linear *GRAMD4* mRNA unchanged (Figure 2B). Simultaneously, we generated a *circGRAMD4* overexpression vector, which significantly increased *circGRAMD4* levels in another RCC cell line, A498, where *circGRAMD4* abundance was relatively lower among the RCC cell lines (Figure S3B). In vitro RCC cells, altering the expression of *circGRAMD4* did not significantly impact cell proliferation, migration, apoptosis, or cell cycle (Figure S3C-G). However, a positive correlation was observed between *circGRAMD4* and CD8^+^ T-cell infiltration in the TME based on WGCNA analysis and immunofluorescence staining of CD8A in tumor tissue slices from RCC patients (Figure S2C, H). Surprisingly, despite this correlation, high expression of *circGRAMD4* and increased CD8^+^ T cells infiltration were found to be associated with a poor prognosis in RCC patients (Figure S2J, K). This finding aligns with previous literature, which reported that a higher proportion of tumor-infiltrating CD8^+^ T cells in RCC patients are associated with unfavorable outcomes [13]. Therefore, we hypothesize that *circGRAMD4* expression may lead to an exhausted and dysfunctional phenotype in tumor-infiltrating CD8^+^ T cells. To investigate this, we examined CD8^+^ T cell depletion-related markers in RCC tissue slices from patients using multiplexed fluorescence immunohistochemistry (mfIHC). The results revealed that PDCD1/PD-1 and LAG3 exhibited a high level of surface expression on CD8^+^ T cells in tumor tissues with elevated *circGRAMD4* expression. In contrast, only minimal expression of depletion-related markers was observed on CD8^+^ T cells in tumor tissues with low levels of *circGRAMD4* expression (Figure 2C). Following this, we co-cultured CD8^+^ T cells with RCC cells and conducted a T cell-induced cytotoxicity test to show that reducing *circGRAMD4* made RCC cells more susceptible to CD8^+^ T cell-induced cell death, while increasing *circGRAMD4* had the opposite outcome (Figure 2D, S4A). Subsequently, we investigated if *circGRAMD4* influenced the immunosuppressive properties of tumor cells on CD8^+^ T cells. Detection of killer cytokines IFNG/IFN-γ, TNF/TNF-α, and GZMB secreted by CD8^+^ T cells revealed that reduced *circGRAMD4* expression relieved tumor cell-induced immunosuppression, whereas increased *circGRAMD4* expression suppressed the production or release of these immune factors (Figure 2E, S4B). Next, we specifically knocked down the *circGRAMD4* homologous circRNA *mmu_circ_0005931* (*circGramd4*) in the Renca mouse RCC cell line (Figure S4C). In vitro, the proliferation of Renca cells is not significantly affected by the absence of *circGramd4* (Figure S4D-E). In vivo experiments showed that *circGramd4* deficiency (*shcircGramd4*) did not impact tumor development in T-cell-deficient nude mice (Balb/c-nu) compared to control cells (shnc) (Figure S4F-H); interestingly, in immune-normal Balb/c mice, *circGramd4* deficiency significantly inhibited tumor growth (Figure 2F-H). To assess whether *circGramd4* deficiency enhances T cell cytotoxicity in vivo, flow cytometry was used to assess the functionality of CD8^+^ T cells infiltrating the tumor. The levels of cytotoxic factors synthesized or secreted by these tumor-infiltrating CD8^+^ T cells and indicators of CD8^+^ T cells exhaustion were then measured. The results revealed that CD8^+^ T cell effector function was enhanced in the *shcircGramd4* group, and the expression of immunosuppressive molecules was reduced (Figure 2I, S4I). In addition, we also measured the infiltration levels of other immune cells in the TME. The results showed that the infiltration levels of CD8^+^ T cells and tumor-associated macrophages (TAMs) cells were different between the shnc group and *shcircGramd4* group, consistent with the single-cell sequencing analysis results in Figure 1D, but there was no significant difference statistically, which may be related to the short growth time of subcutaneous tumors in mice. No significant difference in the proportion of other immune cells indicating that the effector function of CD8^+^ T cells is the main reason affecting tumor growth (Figure S4I, J). These findings suggest that *circGRAMD4* exerts its influence on tumor growth by regulating the interactions between tumor cells and CD8^+^ T cells, ultimately inhibiting CD8^+^ T cell function.

### *CircGRAMD4* reduces MHC-I levels by activating autophagy

**Figure 3.**
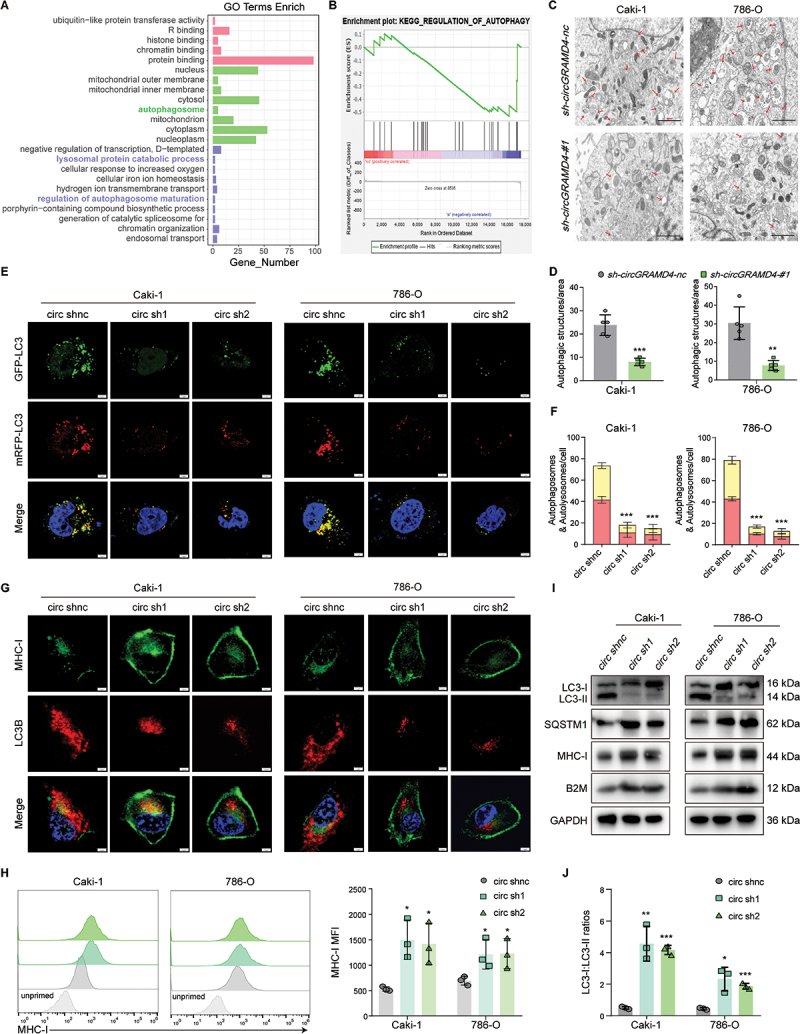
*CircGRAMD4* reduces MHC-I levels by activating autophagy. (A) Gene ontology (GO) analysis on differentially expressed genes in the shnc and *shcircGRAMD4* groups. (B) gene sets enrichment analysis (GSEA) of genes in the shnc and *shcircGRAMD4* groups. (C) Electron micrographs and (D) statistical results indicating a decreased number of autophagosomes and/or autolysosomes (red arrows) in RCC cells treated with *sh-circGRAMD4*. (E) and (F) GFP-mRFP-LC3 labeled caki-1 and 786-O cells were infected with shnc or *sh-circGRAMD4*. Autophagic flux was analyzed by confocal microscopy. Scale bar: 5 μm. (G) Detection of MHC-I and LC3B levels in Caki1 and 786-O cells transfected with shnc or *sh-circGRAMD4* using immunofluorescence assay. Scale bar: 5 μm. (H) Representative images and statistical quantification of the FACS analysis of MHC-I molecules on cell surface. (I) LC3-I:II, SQSTM1, MHC-I, B2M and GAPDH protein expression was measured by western blot. (J) Statistical results of LC3-I:LC3-II. Data are shown as mean± SD; *p < 0.05, **p < 0.01, ***p < 0.001.

Having established the inhibitory effect of high *circGRAMD4* expression in RCC cells on the cytotoxic effect of CD8^+^ T cells, our objective was to elucidate the underlying potential mechanisms. Firstly, we performed transcriptome sequencing on the shnc and *shcircGRAMD4* groups of RCC cells, then analyzed the distinct gene expressions using bioinformatics. Through gene ontology (GO) analysis and gene sets enrichment analysis (GSEA), we found that *circGRAMD4* expression levels may impact the regulation of cellular autophagy in RCC(Figure 3A,B). Currently, more and more studies have indicated that autophagy is related to tumor immune escape, and inhibiting autophagy can enhance the therapeutic efficacy of ICB [24,26–28]. We performed CD8^+^ T cell exhaustion score and tumor cell autophagy score again on single-cell sequencing data (GSE242299). The results showed that in tissues with high levels of CD8^+^ T cell exhaustion, tumor cells had higher autophagy scores (Figure S5A-C). These results to some extent indicate a correlation between autophagy in tumor cells and effector function/depletion of CD8^+^ T cells. Next, we detected the levels of cellular autophagy after *circGRAMD4* level changed. Electron microscopy showed a decreased number of autophagosomes and/or autolysosomes in RCC cells after knocking down *circGRAMD4* (Figure 3C,D). Next, we transfected RCC cells stably knockdown or overexpressing *circGRAMD4* with mRFP-GFP-LC3 [29], and explored the impact of *circGRAMD4* on autophagy through monitoring changes in fluorescence. Notably, RCC cells with *circGRAMD4* knockdown exhibited a reduction in the number of red and yellow LC3 puncta (Figure 3E-F). Conversely, the number of red and yellow LC3 puncta was observed to increase following *circGRAMD4* overexpression (Figure S5D-E). The results of this study provide confirmation that *circGRAMD4* activates autophagy in RCC cells. Numerous studies have demonstrated that autophagy can hinder the MHC-I antigen presentation pathway or lead to MHC-I degradation, ultimately promoting tumor immune escape [24,30]. However, there is a paucity of literature on the relationship between autophagy and MHC-I in RCC. Firstly, we queried the transcriptome sequencing data and found that the transcriptome level of MHC-I related genes did not change significantly after *circGRAMD4* was knocked down, so we speculated that *circGRAMD4* might affect the protein level of MHC-I molecules. Next, immunofluorescence and flow cytometry were used to detect the levels of MHC-I molecules in RCC cells. In the *circGRAMD4* knockout group associated with decreased autophagy levels, MHC-I molecules level was higher than that in the control group (Figure 3G-H). Conversely, in the *circGRAMD4* overexpression group (associated with increased autophagy levels), there was observed a reduction in the level of MHC-I molecules (Figure S5F-G). Subsequently, we performed western blot to assess the expression of key autophagy proteins and MHC-I-related proteins. The results demonstrated a significantly increased LC3-I:LC3-II ratio, MHC-I, B2M/β-2 M, and SQSTM1 in the *circGRAMD4* knockdown group in comparison with the control group cells (Figure 3I-J). In contrast, in the *circGRAMD4* overexpression group, there was a decrease in the LC3-I:LC3-II ratio, and the MHC-I, B2M, and SQSTM1 proteins were significantly downregulated (Figure S5H). These findings indicate that *circGRAMD4* plays a regulatory role in autophagy, which in turn influences the level of MHC-I expression in RCC cells.

### CircGRAMD4 functions by interacting with the RBM4 protein

**Figure 4.**
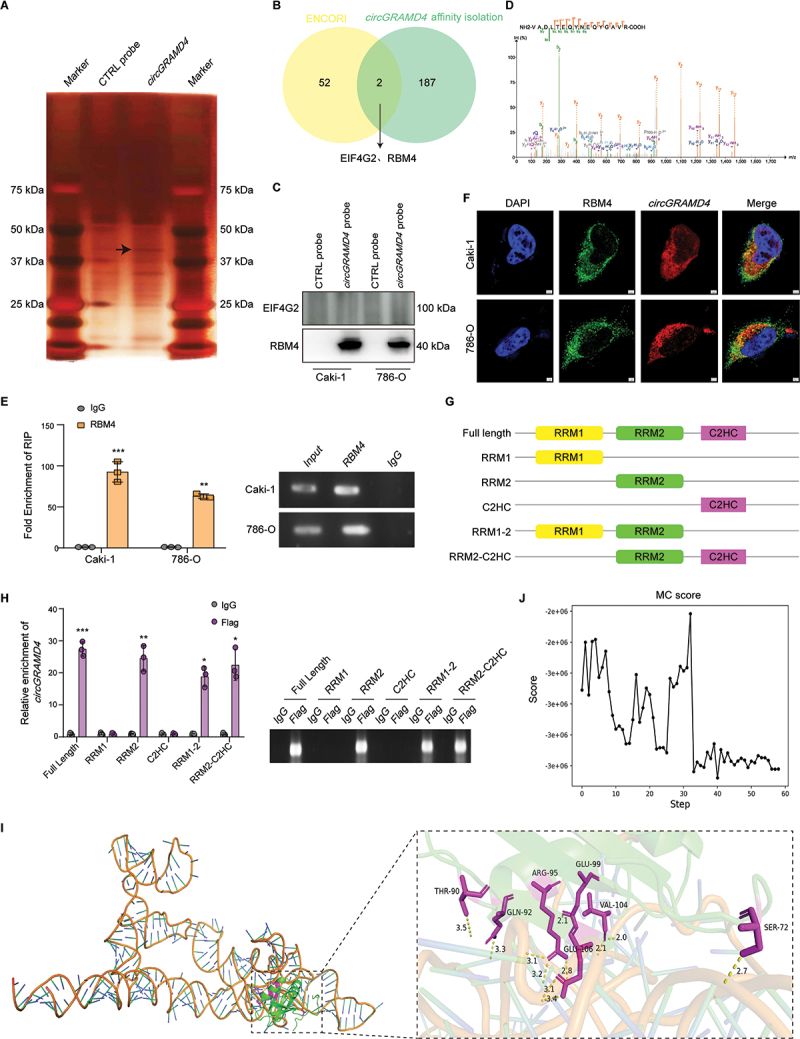
*CircGRAMD4* functions by interacting with the RBM4 protein. (A) RNA affinity-isolation assay with *circGRAMD4* probe and control probe in 786-O cells, followed by silver staining. (B) Overlap of proteins obtained from mass spectrometry and ENCORI database analysis. (C) Western blot after RNA affinity isolation analysis revealed that RBM4 was affinity isolated by *circGRAMD4* probe. (D) Mass spectrometric secondary structure of RBM4 protein. (E) RIP experiments showed interaction between *circGRAMD4* and RBM4 in caki-1 and 786-O cell lines. (F) IF-FISH assays showed that *circGRAMD4* and RBM4 colocalized in the caki-1 and 786-O cells’ cytoplasm. (G) Schematic shows the structure of the RNA-binding domain in RBM4 protein and the structure of RBM4 truncation. 3× flag tag is connected to the C-end of each truncated body. (H) Relative enrichment identified by RIP assay represents RBM4 truncated correlated *circGRAMD4* levels. (I) 3D structure of *circGRAMD4* and the optimal 3D structural diagram of the docking model between *circGRAMD4* and RBM4 (RRM2 domain). (**J**) refinement of the best docked *circGRAMD4-*RBM4 (RRM2 domain) model showing MC score vs steps of simulation. Data are shown as mean± SD; *p < 0.05, **p < 0.01, ***p < 0.001.

In order to uncover the true function of *circGRAMD4* in RCC, we initially utilized TransCirc, a dedicated database offering extensive proof backing the translational capability of circRNAs, to assess if *circGRAMD4* possessed translational capacity [31]. Despite the identification of ORF and IRES sequences and ribosome/polysome profiling, proteomics evidence from mass spectrometry (MS) and analysis of translation initiation sites did not confirm the translation capability of *circGRAMD4*. Recent research reports indicate a growing possibility that circRNAs can engage with proteins and potentially impact the development of different types of cancers [17]. Therefore, we conducted RNA affinity isolation followed by MS to discover proteins that could potentially bind to *circGRAMD4*. Through this approach, we identified a total of 189 proteins that specifically interact with *circGRAMD4* (Data S3, S4). Additionally, we utilized the ENCORI database (https://rnasysu.com/encori/) to forecast potential RNA-binding proteins (RBPs) that might bind with *circGRAMD4*, resulting in the identification of 54 proteins (Figure 4A, B). It was found that proteins EIF4G2 and RBM4 coexist in both MS analysis and ENCORI database prediction results. Next, we further validated using western blot experiments and found that RBM4, rather than EIF4G2, was significantly enriched in the RNA affinity isolation product of *circGRAMD4* probe (Figure 4C). Therefore, we thought that *circGRAMD4* may play its role mainly by binding to RBM4 protein. The molecular mass of the RBM4 protein is about 40 kDa, which is consistent with the obvious differential band position in the silver staining results. To verify the reliability of the MS results, secondary spectral analysis was also performed, which further supported the findings (Figure 4D). Hence, we hypothesize that the interaction between *circGRAMD4* and the RBM4 protein may play a crucial role in the development of RCC. Subsequently, we conducted RNA immunoprecipitation (RIP) experiments to further validate the interaction between *circGRAMD4* and RBM4 (Figure 4E). Next, we confirmed the colocalization of *circGRAMD4* and RBM4 in the cytoplasm through immunofluorescence and fluorescence in situ hybridization (IF-FISH) assays (Figure 4F, S5I). Our investigation will now delve into identifying the specific domain of RBM4 responsible for binding to *circGRAMD4*. To achieve this, we consulted the Smart and UniProt databases and generated RBM4 mutants with truncations in individual RNA recognition motif (RRM) domains (Figure 4G). Remarkably, RIP assays demonstrated that the RRM2 domain of RBM4 exhibited a specific interaction with *circGRAMD4* (Figure 4H). We utilized RNA Composer (https://rnacomposer.cs.put.poznan.pl/) to predict the 3D structure of *circGRAMD4* based on its sequence and previously predicted secondary structure (Figure 4I). Subsequently, we performed docking simulations between *circGRAMD4* and the RBM4 RRM2 domain using the NPDock web server [32]. The predicted combination diagram (Figure 4I) and MC score (Figure 4J) both provide evidence supporting the binding of *circGRAMD4* to the RRM2 domain of RBM4. Collectively, our data reveal that RBM4 interacts with *circGRAMD4* through the RRM2 domain.

### RBM4 participates in activating autophagy, degrading MHC-I, and inhibiting CD8^+^ T-cell toxicity

**Figure 5.**
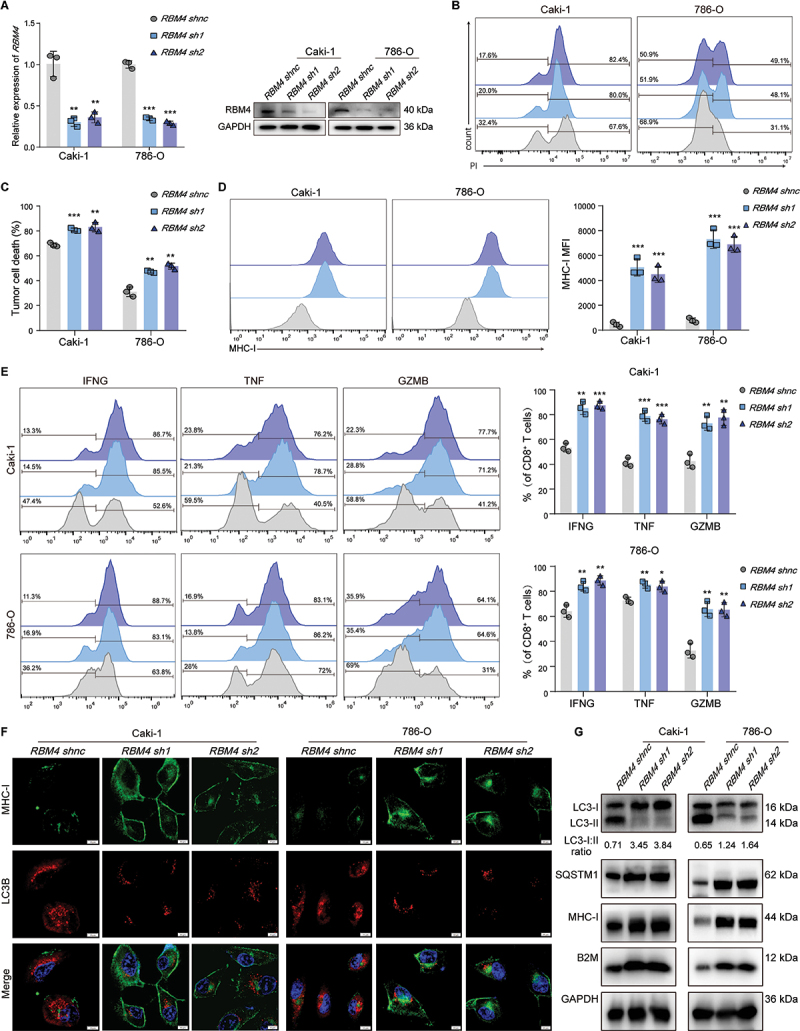
RBM4 participates in activating autophagy, degrading MHC-I, and inhibiting CD8^+^ T cells toxicity. (A) RBM4 knockdown efficiency was measured by qRT-PCR and western blot in caki-1 and 786-O cell lines. (B) Representative flow cytometry images and (C) statistical quantification of the FACS analysis of death rate (PI^+^) of primary kidney tumor cells with or without RBM4 knockdown cocultured with primary kidney tumor-specific CD8^+^ T cells. (D) Representative images and statistical quantification of the FACS analysis of MHC-I molecules on cell surface. (E) Representative images and statistical quantification of the FACS analysis of the percentage of IFNG^+^, TNF^+^, and GZMB^+^ CD8^+^ T cells cocultured with primary kidney tumor cells with or without RBM4 knockdown. (F) Detection of MHC-I and LC3B levels in caki-1 and 786-O cells transfected with shnc or *sh-RBM4* using immunofluorescence assay. Scale bar: 5 µm. (G) LC3-I:II, SQSTM1, MHC-I, B2M and GAPDH protein expression was measured by western blot. Data are shown as mean± SD; *p < 0.05, **p < 0.01, ***p < 0.001.

After demonstrating that RBM4 can directly bind to *circGRAMD4*, we will further investigate whether changes in RBM4 levels affect autophagy and CD8^+^ T cell function in RCC cells. Firstly, the expression of RBM4 was either decreased or increased in renal cancer cells, respectively (Figure 5A, S5J). Flow cytometry analysis showed knockdown or overexpression of RBM4 in vitro RCC cells had no significant effects on cell apoptosis. Next, we conducted CD8^+^ T cell-mediated killing experiments and found that when RBM4 was knocked down, CD8^+^ T cell-mediated renal cancer cell death increased, while when RBM4 was overexpressed, CD8^+^ T cell-mediated renal cancer cell death decreased (Figure 5B-C, S5K-L). We then investigated whether RBM4 affects the immunosuppressive impact exerted by tumor cells on CD8^+^ T cells. The results demonstrated that low expression of RBM4 promoted the expression or secretion of killer cytokines, including IFNG, TNF and GZMB, by CD8^+^ T cells. Conversely, the overproduction of RBM4 resulted in the inhibition of the secretion and expression of these immune effectors (Figure 5E, S5M). Next, we used immunofluorescence and flow cytometry to detect the LC3B and MHC-I levels in RCC cells. In the *RBM4* knockout group, we found a decrease in LC3B levels in RCC cells and an increase in MHC-I molecules levels on the cell surface (Figure 5D,F). Conversely, in the *RBM4* overexpression group, LC3B levels increased and MHC-I molecular levels decreased (Figure SN-O). Additionally, western blot results revealed a notable increase in the ratio of LC3-I:LC3-II in the RBM4 knockdown group in comparison to the control group cells. Notably, MHC-I, B2M, and SQSTM1 were significantly upregulated in this group (Figure 5G). Conversely, in the *RBM4* overexpression group, the proportion of LC3-I:LC3-II, MHC-I, B2M, and SQSTM1 proteins were significantly downregulated (Figure S5P). These observations indicate that RBM4 is involved in regulating both immune responses and autophagy in RCC cells.

### CircGRAMD4 facilitates RBM4 binding and stabilizes NBR1 mRNA

**Figure 6.**
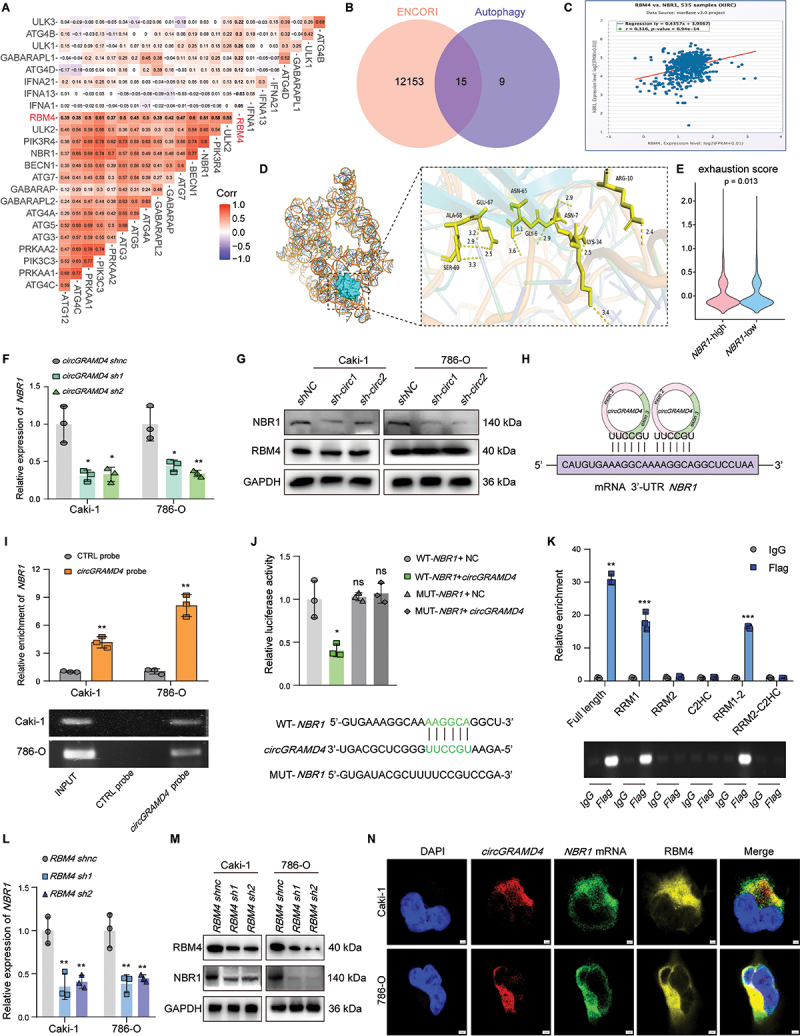
*CircGRAMD4* facilitates RBM4 binding and stabilizes *NBR1* mRNA. (A) Heat map of correlation analysis between RBM4 and genes in autophagy pathway. (B) Overlap of genes in autophagy pathway and candidate mRNA interacting with RBM4 protein predicted by ENCORI database. (C) Correlation analysis between *RBM4* and *NBR1* using KIRC data from the TCGA database. (D) Schematic diagram of *NBR1* mRNA binding to RRM1 domain of RBM4 protein. (E) Violin plot of CD8^+^ T cell exhaustion scores for *NBR1-*high and *NBR1*-low groups (GSE242299). (F)*NBR1* mRNA expression levels was measured by qRT-PCR after *circGRAMD4* knockdown. (G) NBR1 and RBM4 protein levels was measured by western blot after *circGRAMD4* knockdown. (H) Schematic illustration of the binding between *circGRAMD4* and *NBR1* mRNA. (I) RNA affinity-isolation assay showed interaction between *circGRAMD4* and *NBR1* mRNA in caki-1 and 786-O cell lines. (J) Dual-luciferase gene reporter assay showed interaction between *circGRAMD4* and *NBR1* mRNA. (K) Relative enrichment identified by RIP assay represents RBM4 truncated correlated *NBR1* mRNA levels. (L) *NBR1* mRNA expression levels was measured by qRT-PCR after RBM4 knockdown. (M) NBR1 and RBM4 protein levels was measured by western blot after RBM4 knockdown. (N) IF-FISH assays showed that *circGRAMD4*, *NBR1* mRNA and RBM4 protein colocalized in the cytoplasm. Data are shown as mean± SD; *p < 0.05, **p < 0.01, ***p < 0.001.

RBM4 is known as a typical RNA-binding protein and is crucial for maintaining mRNA stability [33]. Based on this knowledge, we hypothesize that the *circGRAMD4*-RBM4 complex may enhance autophagy in RCC cells by safeguarding downstream autophagy-related gene mRNA from rapid degradation. Firstly, we analyzed the data of kidney clear cell carcinoma (KIRC) in TCGA using bioinformatics and found that RBM4 is almost positively correlated with the gene level of autophagy pathway (Figure 6A). In addition, candidate mRNA interacting with RBM4 protein was predicted using the ENCORI database, and 12,168 mRNA were identified, of which 15 genes directly regulate the autophagy pathway (Figure 6B). After reviewing the literature, we found that NBR1 can promote MHC-I to transport to lysosomes through an autophagy dependent pathway, mediating its own degradation [24]. A correlation between expression levels of *RBM4* and *NBR1* in KIRC was observed in the TCGA database (Figure 6C). As forecast by the Alphafold Server website (https://alphafoldserver.com/), we found that *NBR1* mRNA can bind to the RRM1 domain of RBM4 protein (Figure 6D). Then, we used single-cell sequencing data GSE242299 to verify the relationship between *NBR1* and CD8^+^ T cell exhaustion. Based on the expression level of *NBR1* in tumor cells, tumor tissues were divided into high and low expression groups of *NBR1*, and the exhaustion scores of CD8^+^ T cells were calculated for each group. We found that the CD8^+^ T cell dysfunction was more significant in the high expression group of *NBR1*(Figure 6E). Next, we tested whether the expression levels of RBM4 and *NBR1* were affected by changes in *circGRAMD4* expression. The experimental results demonstrated that increased expression of *circGRAMD4* led to elevated mRNA and protein levels of *NBR1* (Figure S6A). Conversely, when *circGRAMD4* expression was downregulated, *NBR1* mRNA and protein levels decreased. Notably, the protein levels of RBM4 remained unaffected (Figure 6F-G). According to the results of actinomycin D experiment, we found that downregulating *circGRAMD4* significantly inhibited *NBR1* mRNA stability (Figure S6B). Through sequence BLAST analysis, we identified a UUCCGU site near the back-splicing junctions of *circGRAMD4* that can bind to the AAGGCA site in the 3’-UTR region of *NBR1* mRNA (Figure 6H). Subsequently, RNA affinity isolation assays were employed to substantiate the interaction between *circGRAMD4* and *NBR1* mRNA (Figure 6I). Additionally, luciferase reporter genes were constructed for both wild-type *NBR1* and mutant *NBR1*. According to the dual-luciferase gene reporter assay, overexpression of *circGRAMD4* led to a notable reduction in luciferase activity within the WT *NBR1* group. However, there was no discernible effect observed in the MUT *NBR1* group (Figure 6J). Same as the predicted result, RIP assays also demonstrated that the RRM1 domain of RBM4 specifically binds to *NBR1* mRNA (Figure 6K). Additionally, we investigated whether alterations in RBM4 expression impact *NBR1* levels. Our findings revealed that decreased RBM4 expression led to reduced mRNA and protein levels of *NBR1*, whereas RBM4 overexpression resulted in increased *NBR1* mRNA and protein content (Figure 6L, M, S6C). FISH-IF assays showed that *NBR1* mRNA, RBM4 protein as well as *circGRAMD4* were colocalized in the cytoplasm (Figure 6N, S6D). Next, qRT-PCR and WB experiments showed that the overexpression of RBM4 protein could significantly increase the expression level of *NBR1*, but this effect was significantly weakened when *circGRAMD4* was knocked down (Figure S6E-G). Knockdown of *circGRAMD4* markedly reduced the *NBR1-*RBM4 RNA-protein interaction as shown in the RIP assays, whereas overexpression of *circGRAMD4* significantly increased the enrichment of *NBR1* in RBM4 immunoprecipitated fractions (Figure S6H). These findings demonstrate that *circGRAMD4* plays a critical role in promoting the interactions between RBM4 and *NBR1*, and enhances the mRNA stability of *NBR1* through the formation of a *circGRAMD4*-RBM4*-NBR1* RNA-protein ternary complex.

### CircGRAMD4 and RBM4 affect autophagy and MHC-I degradation by affecting the expression of NBR1, and promote immune escape

**Figure 7.**
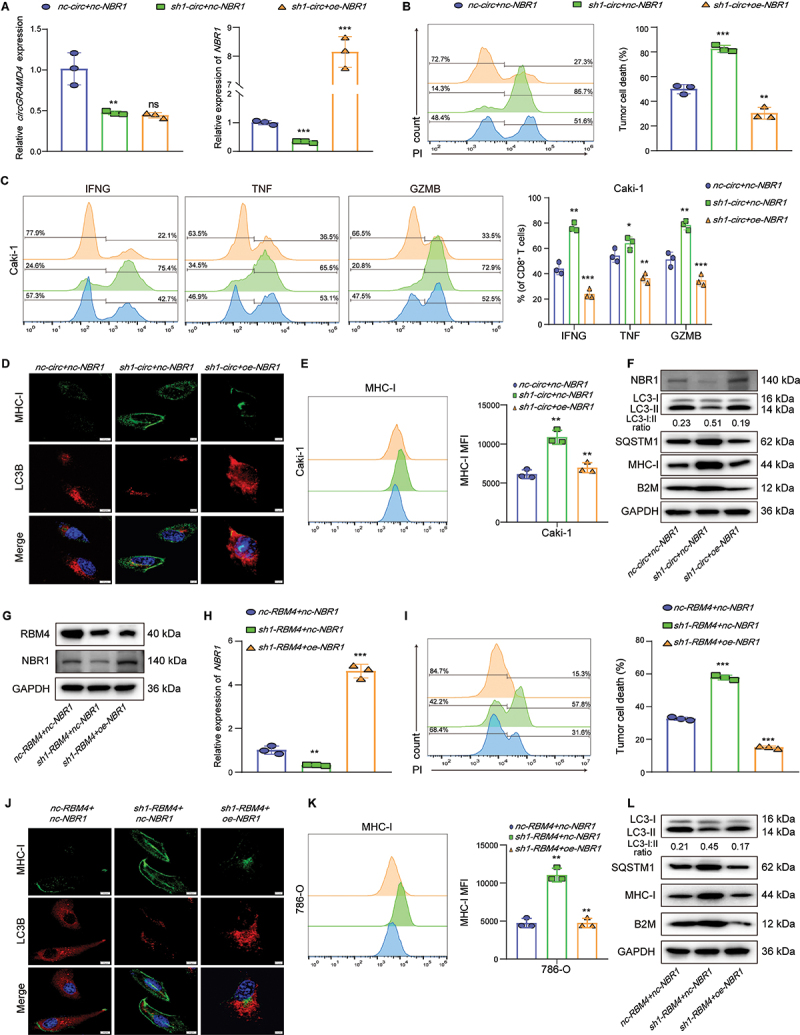
*CircGRAMD4* and RBM4 affect autophagy and MHC-I degradation by affecting the expression of *NBR1*, and promote immune escape. (A) qRT-PCR assay assessing the expression level of *circGRAMD4* and *NBR1* in caki-1 cells. (B) Representative flow cytometry images of death rate (PI^+^) of caki-1 cells cocultured with primary kidney tumor-specific CD8^+^ T cells. (C) Representative images and statistical quantification of the FACS analysis of the percentage of IFNG^+^, TNF^+^, and GZMB^+^ CD8^+^ T cells cocultured with caki-1 cells. (D) Detection of MHC-I and LC3B levels in caki-1 cells using immunofluorescence assay. Scale bar: 5 μm. (E) Representative images and statistical quantification of the FACS analysis of MHC-I molecules on cell surface. (F) NBR1, LC3-I:II, SQSTM1, MHC-I, B2M and GAPDH protein expression was measured by western blot. (G) Western blot assay assessing the expression level of RBM4 and NBR1 in 786-O cells after *RBM4* and/or *NBR1* changed. (H) qRT-PCR assay assessing the expression level of *NBR1* in 786-O cells. (I) Representative flow cytometry images of death rate (PI^+^) of 786-O cells cocultured with primary kidney tumor-specific CD8^+^ T cells. (J) Detection of MHC-I and LC3B levels in 786-O cells using immunofluorescence assay. Scale bar: 5 μm. (K) Representative images and statistical quantification of the FACS analysis of MHC-I molecules on cell surface. (L) LC3-I:II, SQSTM1, MHC-I, B2M and GAPDH protein expression was measured by western blot. Data are shown as mean± SD; *p < 0.05, **p < 0.01, ***p < 0.001.

To investigate the effects of *circGRAMD4* binding to *NBR1* mRNA on cell function, we modulated the expression levels of *circGRAMD4* and *NBR1* in the Caki-1 cell line using *sh-circGRAMD4* and *oe-NBR1*, respectively. Subsequently, we assessed *circGRAMD4* and *NBR1* mRNA levels in the three experimental groups using qRT-PCR (Figure 7A). Flow cytometry showed that there was no significant change in the apoptosis level of tumor cells not co-cultured with CD8^+^ T cells. However, the results of the CD8^+^ T cell-mediated killing assay revealed that *circGRAMD4* knockdown led to an increase in CD8^+^ T cell-mediated Caki-1 cell death, however, the effect could be rescued by overexpression of *NBR1*(Figure 7B). Subsequently, we employed flow cytometry to assess the levels of cytotoxic cytokines secreted by CD8^+^ T cells co-cultured with Caki-1 cells. The results revealed that *NBR1* overexpression in Caki-1 cells could reduce the expression of CD8^+^ T cell killing factors (including IFNG, TNF and GZMB) induced by *circGRAMD4* knockdown (Figure 7C). Knockout of *circGRAMD4* in Caki-1 can reduce autophagy and increase cell surface MHC-I level, while overexpression of *NBR1* can restore this effect (Figure 7D-E). Western blot results showed that *NBR1* overexpression could restore the protein expression changes caused by *circGRAMD4* knockout (Figure 7F). These results suggest that *NBR1* can restore RCC autophagy, induce MHC-I degradation, and then affect the function of CD8^+^ T cells caused by *circGRAMD4* knockout.

Next, we investigate the effects of RBM4 and *NBR1* mRNA binding on cell function. We manipulated the expression levels of RBM4 and *NBR1* in the 786-O cell line using *sh-RBM4* and *oe-NBR1*, respectively. Subsequently, we employed western blot and qRT-PCR in order to assess the levels of RBM4 and *NBR1* within the three experimental groups (Figure 7G-H). Similarly, there was no significant change in the apoptosis level of tumor cells not co-cultured with CD8^+^ T cells. While the results of CD8^+^ T cell-mediated killing assay showed that *RBM4* knockdown led to an increase in CD8^+^ T cell-mediated 786-O cell death, however, the effect could be rescued by overexpression of *NBR1* (Figure 7I). Similarly, *NBR1* overexpression in 786-O cells could reduce the expression of CD8^+^ T cell killing factors induced by *RBM4* knockout (Figure S6I). Knockout of *RBM4* in 786-O can reduce autophagy and increase cell surface MHC-I level, while overexpression of *NBR1* can restore this effect (Figure 7J-K). Western blot results showed that *NBR1* overexpression could restore the protein expression changes caused by *RBM4* knockout (Figure 7L). These results suggest that *NBR1* can restore RCC autophagy, induce MHC-I degradation, and then affect the function of CD8^+^ T cells caused by *RBM4* knockout.

Finally, to prove that the increased cancer cell death upon knockdown of *circGRAMD4*, RBM4 and *NBR1* is mediated by increased MHC-I expression on cancer cells, cell surface MHC-I was depleted by knocking down *B2M*, a critical component of the MHC-I complex (Figure S7A-B). Then we conducted rescue experiments on tumor cells that had knocked out *circGRAMD4*, *RBM4* and *NBR1* by knocking out *B2M* again to reduce the expression level of MHC-I on the cell surface (Figure S7C). We found that the elevated MHC-I levels on tumor cells surface after knocking out *circGRAMD4*, *RBM4*, and *NBR1* decreased again with the knockout of *B2M* (Figure S7D), and the corresponding changes in cancer cell death (Figure S7E) and CD8^+^ T cytokine levels also occurred (Figure S7F). These results can prove that the increase in cancer cell death after knocking out *circGRAMD4*, *RBM4* and *NBR1* is mediated by the increased expression of MHC-I on cancer cells.

### Knocking down circGRAMD4 helps to synergistically enhance the efficacy of PDCD1 therapy in PDX models

**Figure 8.**
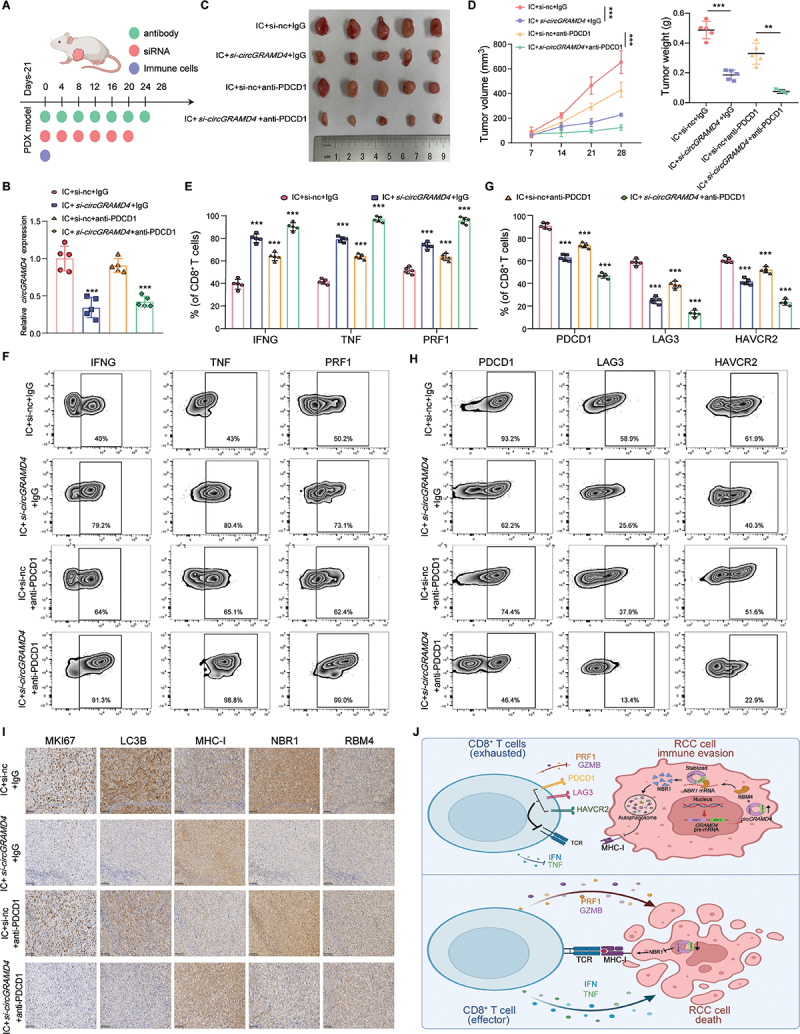
Knocking down *circGRAMD4* helps to synergistically enhance the efficacy of PDCD1 therapy in PDX models. (A) Schematic diagram showing that RCC PDX mice were treated with adoptive T cell transfer, antibody and siRNA at the indicated time points. In the figure, −21 indicates the day of subcutaneous inoculation of RCC PDXs. (B) qRT-PCR analysis showing *circGRAMD4* expression levels in tumor tissues from PDX mice with the indicated treatments. (C) Images of the collected subcutaneous xenograft tumors from NCG mice. (D) Left: record the tumor volume of tumor bearing NCG mice every 7 days after IC transfer. Right: eventual weights of subcutaneous xenograft tumors. Flow cytometry analysis and statistical results of killing factor (E and F) and immunosuppressive molecules (G and H) in CD8^+^ T cells isolated from PDX tumors in NCG mice. (I) Representative IHC staining images of MKI67, LC3B, MHC-I, NBR1 and RBM4 in tumors from PDX mice with the indicated treatments. Scale bar: 100 μm. (J) Schematic diagram of the mechanism of *circGRAMD4* mediated tumor immune escape in RCC. Data are shown as mean± SD; *p < 0.05, **p < 0.01, ***p < 0.001.

Building upon our previous observations, our objective was to investigate the potential of *circGRAMD4* in human tumors as a means of enhancing the effectiveness of RCC immunotherapy. In order to replicate the TME of RCC more accurately, we employed a preclinical model involving the implantation of RCC patient-derived xenografts into immunocompromised NCG mice, followed by the transfer of tumor-specific CD8^+^ T cells [34,35]. To study the effect of inhibiting *circGRAMD4* on the efficacy of anti-PDCD1 immunotherapy in a model of a humanized immune system, a particular siRNA directed against *circGRAMD4* was utilized (Figure 8A). In PDX tumor tissues, *circGRAMD4* was effectively reduced by treatment with siRNA (Figure 8B). Additional examination revealed that while *circGRAMD4* siRNA and anti-PDCD1 single treatment both hindered tumor progression, the combination of *si-circGRAMD4* and PDCD1 inhibition demonstrated a more pronounced effect in reducing tumor burden than either *si-circGRAMD4* or anti-PDCD1 alone (Figure 8C-D). Furthermore, the effector function of CD8^+^ T cells was significantly augmented by the combination therapy, as demonstrated by flow cytometry analysis of killer cytokines and exhaustion-related indicators (Figure 8E-H). As anticipated, immunohistochemistry staining of PDX tumor tissues showed that treatment with *si-circGRAMD4* resulted in reduced levels of MKI67/Ki67, LC3B, and NBR1 proteins, while increasing MHC-I protein levels. Interestingly, there was no significant impact on the expression of RBM4 protein (Figure 8I). Overall, these results suggest that blocking *circGRAMD4* can improve the effectiveness of PDCD1 blockade, thereby offering a promising avenue for the advancement of cancer immunotherapy outcomes in RCC.

## Discussion

Researchers have shown that tumor-infiltrating lymphocytes are associated with the improvement of prognosis of many cancer patients [5,36,37]. CD8^+^ T cells are crucial for tumor immune monitoring. Their presence in TME is often considered a good prognostic indicator and may indicate a response to ICB therapy. However, recently published RCC proteomic studies have revealed that GP1 subtypes of RCC, despite exhibiting robust immune activity, are correlated with unfavorable clinical outcomes [13,38,39]. In addition, RCC appears to employ a distinct immune escape mechanism by impairing the cytotoxic T cell killing function rather than directly repelling T cells. This evasion strategy allows RCC to avoid immune system attacks [40]. Notably, this dysfunction in T cells can significantly impact a patient’s response to ICB therapy. While anti-PDCD1 therapy can restore T cells with early functional impairment, it may prove ineffective for T cells with severe functional deficits [41,42]. Currently, the specific immune suppression and immune evasion mechanisms present within the RCC TME remain poorly understood.

In the present study, we utilized WGCNA and CIBERSORT-based immune infiltrate estimation to identify potential circRNAs that may influence antitumor immunity in RCC. Notably, we discovered *circGRAMD4* as a significantly upregulated circRNA that positively correlates with CD8^+^ T cells infiltration and poorer prognosis. Importantly, our investigations confirmed that *circGRAMD4* is predominantly expressed in RCC cells rather than immune cells. Furthermore, when CD8^+^ T cells were co-cultured with primary RCC cells lacking *circGRAMD4*, they exhibited enhanced cytotoxicity and anti-tumor function. Using immunodeficient mouse models, we observed that *circGRAMD4* depletion inhibited tumor growth and enhanced antitumor immunity. These results indicate that *circGRAMD4* has immunosuppressive effects in RCC. Through transcriptome sequencing analysis, it was found that high expression of *circGRAMD4* can activate the autophagy pathway. Nowadays, a large number of studies indicate a complex relationship between immune escape and autophagy in tumor cells [43,44]. In tumor immune evasion, autophagy may serve the following roles: 1) Autophagy can induce degradation of antigen-presenting molecule MHC-I and immune checkpoint protein CD274/PD-L1 in cancer cells [24,25,45]; 2) Autophagy-related genes can enhance the anticancer effect of immune checkpoint inhibitors by increasing the sensitivity of tumor cells to T-cell cytotoxicity [46,47]; 3) Various stress signals, such as metabolic stress, hypoxia, redox stress, and immune signaling, can induce autophagy in tumor cells. These stress signals play a pivotal role in shaping the TME [43,48]. In our experimental findings, we observed that suppressing *circGRAMD4* reduced autophagy and led to elevated MHC-I expression levels.

Over the past few decades, circRNAs have been recognized for their roles in biological processes via a number of established mechanisms. circRNAs primarily found in the cytoplasm are believed to function as miRNA or protein sponges. Even certain circRNAs can be translated under specific circumstances [49]. In addition, it was proposed that circRNA may play a guiding role in promoting specific connections between RBP and subsequent RNA sequences, forming RNA protein ternary complexes to stabilize mRNA in various types of tumors [50,51]. Our MS analysis revealed significant enrichment of the conventional RBP, RBM4, in the *circGRAMD4* affinity-isolated complex. RBM4 is a common RNA-binding protein that can regulate intracellular signaling pathway activity by affecting mRNA stability and translation efficiency [33,52,53]. In our research, we predicted the three-dimensional structure of *circGRAMD4*. NPDock predicts that *circGRAMD4* can bind to the RRM2 domain of RBM4. We constructed a protein truncation based on the RNA-binding domain of RBM4, and verified through RIP experiments that RBM4 protein can bind to *circGRAMD4* through RRM2. Given that *circGRAMD4* influences cellular autophagy, we hypothesize that its interaction with the RBM4 protein may enhance the expression of genes associated with the autophagy pathway. Based on database predictions and experimental results, we have verified that *circGRAMD4* and RBM4 proteins can bind to the mRNA of *NBR1*, respectively. The interaction of the three components can lead to the formation of an RNA-protein ternary complex, enhancing the stability of *NBR1* mRNA and consequently promoting autophagy. In conclusion, these findings provide insights into the potential molecular mechanisms underlying autophagy overactivation and immune suppression within the TME of RCC. In the TME, elevated *circGRAMD4* expression in tumor cells positively correlates with the proportion of infiltrating CD8^+^ T cells. However, this increased *circGRAMD4* expression can activate MHC-I selective autophagy within tumor cells, leading to reduced MHC-I levels. CD8^+^ T cells depend on tumor-specific peptides displayed on the surface of MHC-I molecules for recognition and targeting of tumor cells. When MHC-I expression decreases, tumor cells can evade immune surveillance, leading to tumor growth and metastasis [54]. Persistent low antigen stimulation can drive CD8^+^ T cells to differentiate into depleted precursor T cells/T_pex_, which possess self-renewal capabilities and memory response functions. However, under continuous antigen stimulation, they may further differentiate into depleted terminal T cells/T_ex_, exhibiting functional abnormalities and depletion. Exhausted CD8^+^ T cells typically exhibit a range of exhaustion-related markers, including PDCD1, ENTPD1/CD39, HAVCR2/TIM3, and LAG3. The expression of these markers is closely correlates with the recognition capacity of CD8^+^ T cells responsive to tumors [55]. Studies suggest that inhibiting autophagy in combination with ICB monotherapy has a synergistic impact [24,56]. In our study, utilizing a preclinical PDX model specific to CD8^+^ T cells, a combination therapy involving the inhibition of *circGRAMD4* and blockade of PDCD1 was found to exhibit a synergistic effect compared to PDCD1 blockade alone. The inhibition of *circGRAMD4* reprograms the TME and renders tumors more responsive to PDCD1 blockade.

## Materials and methods

### Patients and specimens

This study utilized patient samples collected from the Department of Urology at the First Affiliated Hospital of Sun Yat-sen University in Guangzhou, China. The research protocol received approval from the Medical Ethics Committee of the First Affiliated Hospital of Sun Yat-sen University (Approval No. [2023]943). All cases undergo regular follow-up, documenting any recurrence and/or metastasis. Patients provided written consent, and the study adhered to the guidelines outlined in the Helsinki Declaration.

### Bioinformatics

Weighted gene coexpression network analysis (WGCNA) was utilized to identify modules linked to CD8^+^ T cells infiltration. CIBERSORT was utilized to calculate the percentage of CD8^+^ T cells that had infiltrated 5 pairs of RCC tumor tissues and identify circRNAs that potentially promoted CD8^+^ T cells infiltration in RCC tissues. The Seurat R package (v 4.0) was used to analyze the single-cell sequencing gene expression matrices. Genes expressed in more than 0.1% of the data and cells with over 300 detected genes were selected for further analysis. Cells with more than 20% of UMIs derived from the mitochondrial genome were excluded. After removing low-quality cells, the gene expression matrices were normalized and scaled for each gene across all cells. They were then integrated, scaled, and clustered in low-dimensional space using the RunUMAP function with default settings. The “AddModuleScore” function of the Seurat package was used to calculate the mean expression of genes in specific gene sets. Six inhibitory marker genes for CD8^+^-exhausted T cells (PDCD1, CTLA4, HAVCR2, LAG3, TOX, and ENTPD1) were used to calculate the exhaustion score. ENCORI (https://rnasysu.com/encori/) was used to predict candidate RNA-binding proteins (RBPs) adsorbed by *circGRAMD4* and candidate mRNA binding to RBM4. The data used for comparing differences and relationships were transformed using the log2(TPM +1) method. Datasets were gathered and processed, examined, and displayed using R version 4.3.2. The corresponding author can provide all the code that supports the study’s conclusions upon request.

### Cell culture

In this study, we obtained the immortalized renal epithelial cell line HK-2, as well as several human RCC cell lines (A498, 769P, 786-O, and Caki-1), along with the mouse RCC cell line Renca, from the American Type Culture Collection. Additionally, we acquired the 293T cell line derived from human embryonic kidney from the Cell Bank of the Chinese Academy of Sciences in Shanghai, China. The HK-2 cell line was cultured in Keratinocyte Serum-free Medium (K-SFM, Gibco 17,005,042), while MEM Medium (Gibco, C11095500BT) was used for the A498 cells. The 769P and 786-O cell lines were grown in RPMI-1640 medium (Gibco, C11875500BT), and McCoy’s 5A Medium (Gibco 16,600,082) was used for the Caki-1 cell line. The 293T cells were cultured in DMEM (Gibco, C11995500BT). The media were supplemented with 1% penicillin-streptomycin (Biosharp, BL505A) and 10% FBS (ExCell Bio, FSD500). All cells were maintained at 37°C in an environment with 5% carbon dioxide. The vendors conducted STR profiling on all cell lines and regularly checked for mycoplasma infection.

### Agarose gel electrophoresis

Agarose (Biofroxx, 1110GR100) gels (1.2%) were made with 1×Tris-acetate (TAE, Biosharp, BL533A) buffer. The DNA underwent electrophoresis at a voltage of 140 for a duration of 25 min, followed by visualization of the bands using an ultraviolet gel imaging system (UVP GelStudio PLUS touch, Germany).

### qRT-PCR

First, total RNA was extracted from the cells using Trizol reagent (Invitrogen 15,596,018), and the RNA concentration was measured using a Nanophotometer (IMPLEN, Germany). Next, we balanced the concentrations of each RNA sample and reverse transcribed them into cDNA using 4× EZscript Reverse Transcription Mix II (EZBioscience, EZB-RT2GQ). The resulting cDNA, along with forward and reverse primers, 2× Color SYBR Green qPCR Master Mix (EZBioscience, A0012-R2) and RNA free water were added to either a 96-well plate or a 384-well plate following the provided instructions. Finally, PCR reactions were conducted using the QuantStudio real-time PCR system (Thermo Fisher Scientific, USA). The primer sequences used in this experiment are detailed in Table S1.

### RNase R treatment

First, extract total RNA from the cells and measure the nucleic acid concentration. Next, treat the RNA with 3 U/μg RNase R (Lucigen, RNR07250) at 37°C for 15 min. Finally, assess the expression levels of both *circGRAMD4* and linear *GRAMD4* using qRT-PCR.

### Actinomycin D treatment

Cells were seeded into a six-well plate, and once they adhered to the bottom and reached approximately 60% density, we treated them with 2 μg/mL of actinomycin D (MCE, HY-17559) was used to treat cells for 6, 12, 18 and 24 h. Following treatment, we extracted total RNA from each cell group using Trizol (Invitrogen 15,596,026). We then employed RT-qPCR to assess the expression levels of both circRNA and linear RNA at different time points, allowing us to determine their respective half-lives.

### Nuclear and cytoplasmic separation experiment

Cellular components were isolated into nuclear and cytoplasmic fractions with the assistance of a nucleus and cytoplasm separation kit (Bestbio, BB-36021-2). Approximately 5–10 × 10^6^ cells were centrifuged for 2–3 min (4°C, 500 × g) and then washed twice using cold PBS (Gibco, C10010500BT). Extract liquid A (200 μL) was added to the cell precipitate and incubate in a shaking table at 4°C for 30 min. Then, centrifuge at 1200 × g for 5 min using a cooled centrifuge at 4°C. The resulting supernatant is the cytoplasmic portion, while the precipitate is the nuclear portion.

### Fluorescence in situ hybridization (FISH) assay

We performed the FISH experiment using a kit from GenePharma (F21401/50). Specifically, Cy3-labeled *circGRAMD4* and *RNA 18S* probes, along with a FAM-labeled *NBR1* mRNA probe, were synthesized by GenePharma. The step-by-step process involved inoculating cells onto a glass dish, fixing them with 4% paraformaldehyde (Beyotime, P0099) once adhered and permeabilizing with 0.1% Triton X-100 (Beyotime, P0096), adding pre-hybridization solution dropwise, incubating at 37°C for 1 h, and then hybridization solution containing the probes was added to the cells overnight hybridization at 37°C, subsequent washing with SSC (Beyotime, ST462) the following day, staining the cell nucleus with DAPI (Biosharp, BL105A), sealing the cells with an anti-fluorescence quenching agent, and finally capturing images using a laser scanning confocal microscope (OLYMPUS/FV3000, Japan).

## Gene silencing or overexpression

To silence genes, the siRNAs were transfected into cells using Lipo2000 (Invitrogen 11,668,019). ShRNAs were transfected using polybrene (Merck Millipore, TR-1003-G). After an 8-h incubation period, change the cell culture medium and incubate for another 48 h. To induce gene overexpression, cells were transfected with overexpression plasmids using Lipo3000 (Invitrogen, L3000–015) and with lentiviral vectors for overexpression analysis using polybrene (Merck millipore, TR-1003-G).

### Isolation of CD8^+^ T cells and co-culture with tumor cells

Healthy donor PBMCs were obtained through density gradient centrifugation using Lymphocyte Separation Medium (Biolegend 7,111,011). Human CD8^+^ T cells were isolated using a CD8^+^ T cell isolation Kit (IPHASE, 071A103.12) following the manufacturer’s protocol. After adjusting the cell density to 1 × 10^6^ cells/mL, CD8^+^ T cells were stimulated with CD3 (PeproTech 05,121-25-500) and CD28 (PeproTech 10,311-25-500) activator and cultured in 1640 complete medium (GIBCO, C11875500BT) containing 10% FBS (ExCell Bio, FSD500), 1% penicillin- streptomycin (Biosharp, BL505A) and 100 U/mL IL2 (PeproTech, 200-02-100) and 27.5 μM 2-mercaptoehanol (EcoTop Bio, ED-9153), and the solution was changed every 2–3 days. Then the tumor cells and CD8^+^ T cells were co-cultured in the ratio of 1:4 for 24–48 h, and the cells were collected. The tumor cell death and CD8^+^ T cell killing factor were detected by flow cytometry.

### Flow cytometry assay

To identify cell surface antigens, cells were stained with PE anti-human *HLA-A,B,C* (Biolegend 311,405), AF700 anti-human PTPRC/CD45 (Biolegend 368,513), FITC anti-human CD3 (Biolegend 300,305), BV605 anti-human CD8 (Biolegend 344,741), Zombie Aqua™ Fixable Viability Kit (Biolegend 423,101), AF647 anti-human PDCD1/PD-1 (Biolegend 367,419), BV-421 anti-human LAG3 (Biolegend 369,313), PE anti-human HAVCR2/TIM3 (Biolegend 345,005), AF700 anti-mouse PTPRC/CD45 (Biolegend 103,127), APC-Cy7 anti-mouse PTPRC/CD45 (BD 557,659), PE anti-mouse CD3 (Biolegend 100,205), FITC anti-mouse CD8 (BD 557,085), FITC anti-mouse CD4 (Biolegend 100,509), AF647 anti-mouse FOXP3 (Biolegend 320,013), FITC anti-mouse ITGAM/CD11B (Biolegend 101,205), BV421 anti-mouse LY6C/LY6G (GR-1; Biolegend 108,433), PE-Cy7 anti-mouse ADGRE1/F4/80 (Biolegend,123113), APC-Cy7 anti-mouse PDCD1 (Biolegend 135,223), PE-Cy7 anti-mouse LAG3 (Biolegend 125,225), BV421 anti-mouse HAVCR2 (Biolegend 119,723). Zombie Aqua™ Fixable Viability Kit (Biolegend 423,101) was used to judge whether a cell is alive or dead. To detect cytotoxic cytokine production, cells were treated with Cell Activation Cocktail (containing Brefeldin A) from Biolegend (423303) for 6 h at 37°C. Subsequently, the cells were fixed using Fixation buffer (Biolegend 420,801) and permeabilized with Intracellular Staining Perm Wash Buffer (Biolegend 421,002) following the manufacturer’s instructions. Next, cells were stained with PE anti-human IFNG (Biolegend 502,508), BV421 anti-human TNF (Biolegend 502,931), APC anti-human GZMB (Biolegend 372,203), BV650 anti-mouse IFNG Biolegend 505,831BV421 anti-mouse TNF (BD 566,287), PE anti-mouse PRF1/perforin 1 (BD 154,405). The stained cells were analyzed using a CytoFLEX Flow cytometer (Beckman Coulter, USA), and the data were analyzed using FlowJo v10.8.1 software

### Generation of DCs and tumor-specific CD8^+^ T cells

For the generation of DCs, mononuclear cells were obtained from the peripheral blood of HLA-A2^+^ healthy donors and cultured in VIVO medium (Lonza, 04-418Q) supplemented with 100 ng/mL CSF2/GM-CSF (PeproTech, AF-300-03-20) and 30 ng/mL IL4 (PeproTech, AF-200-04-5). The medium and cytokines were changed every 3 days. By the sixth day, mature DCs were stimulated with 10 ng/mL TNF (PeproTech, AF-300-01A-10) for 24 h. Subsequently, the DCs were exposed to tumor lysates from HLA-A2^+^ patients by freeze-thawing with liquid nitrogen for an additional 24 h. Tumor-specific CD8^+^ T cells were produced by isolating CD8^+^ T cells from the peripheral blood of the donors mentioned earlier. To generate tumor-specific T cells, mature DCs were cocultured with isolated CD8^+^ T cells at a 5:1 ratio in VIVO medium (Lonza, 04-418Q) supplemented with 25 IU/mL IL2 (PeproTech, 200-02-100) for 6 days.

### Isolation of primary RCC cells

Separate tumor tissues, wash twice with PBS, then cut the tissues into 1-mm^3^ sized fragments and transfer the tumor fragments into centrifuge tubes. Add collagenase I (0.5–2.5 mg/mL; Stemcell 07,415) and hyaluronidase (0.5–2.5 mg/mL; Stemcell 07,461) into the tubes and shake on a shaker at 37 for 1–2 h. After digestion is complete, take a 70-um cell strainer (Biologix, 15–1070) and place it on a new centrifuge tube. Pour the digested liquid (including cells and cell fragments) into the cell sieve and filter it. After filtration, centrifuge at 200 × g for 5 min and then carefully aspirate the liquid and resuspend with complete culture medium. Transfer the obtained cell suspension to a clean culture dish for cultivation.

### Identifying autophagic flux in cellular systems

Cells were monitored for autophagic flux using a tandem LC3B tagged with mRFP and GFP. Briefly, transducing cells with mRFP-GFP-LC3 lentiviruses for 48 h, mPRF-GFP-LC3 expressed cells were detected by laser scanning confocal microscope (OLYMPUS/FV3000). Manually quantified were the GFP and mRFP puncta counts per cell.

### Immunofluorescence assay

First, inoculate the cells into a confocal culture dish (Biosharp, BS-15-GJM-20EA) and allow them to adhere to the bottom. Next, fix the cells with 4% paraformaldehyde (Beyotime, P0099) for 15 min, followed by permeation using 0.1% Triton X-100 (Beyotime, P0096). Seal the cells with 5% BSA (ABCONE, B24726) for 30 min. Subsequently, incubate the cells overnight with the primary antibody at 4°C. The following day, wash the cells with PBS (Biosharp, BL601A) and incubate them with the secondary antibody at 37°C for 30 min. Finally, stain the cell nuclei with DAPI (Biosharp, BL105A) and capture fluorescence images using an Olympus/FV3000 confocal microscope.

### Western blot analysis

Add 10 uL protease inhibitor (MCE, HY-K0010) to each mL of RIPA lysis buffer (Aladdin, R301900) to prepare cell lysis buffer. Lyse the cells on ice for 10 min with the buffer, then collect the lysis products in an EP tube (NEST 615,601) and use a cell ultrasonic crusher to break the cells for a few seconds to fully lyse. Utilize the BCA protein assay kit (Thermo Fisher Scientific, TFS-23227) to quantify proteins, measuring absorbance at 562 nm with a multi-functional microplate reader (Thermo Fisher Scientific, USA), followed by determining the concentration of individual protein samples. Then the protein sample was loaded into SDS-PAGE gel for protein electrophoresis, and the protein was transferred to PVDF membrane (Merck Millipore, ISEQ00010–1) after electrophoresis. After sealing with 5% skim milk (Beyotime, P0216), the samples were incubated with primary antibodies against LC3B (Cell Signaling Technolgy [CST], 83506S), SQSTM1/p62 (MBL, PM045), MHC-I (abcam, ab134189), B2M (Proteintech 13,511–1-AP), GAPDH (Proteintech 60,004–1-Ig), RBM4 (Proteintech 11,614–1-AP), EIF4G2 (Proteintech 67,428–1-Ig), and NBR1 (Proteintech 16,004–1-AP) at 4°C for at least 8 h. Next, after washing with Tris-buffered saline (BOSTER, AR0031) containing 0.1% Tween 20 (Sigma, V900548), PVDF membranes were incubated with a secondary antibody (Proteintech, SA00001–1, SA00001–2) at room temperature for 1 h. Subsequently, the immunoreactivity was detected using the western blot substrate kit (Tanon, 180–5001) and electrochemiluminescence detection (Amersham ImageQuant 800; GE, USA).

### RNA affinity isolation and silver staining

The RNA affinity-isolation assay was conducted using the Pierce Magnetic RNA-Protein Pull-Down Kit (Thermo Fisher Scientific 20,164) following the manufacturer’s instructions. Each lane of the SDS-PAGE gel contained loaded protein samples. After electrophoresis, the gel was stained using the Fast Silver Stain Kit (Beyotime, P0017S).

### RNA immunoprecipitation (RIP) assays

The EZ-Magna RIP Kit (Merck Millipore, 17–701) was utilized to conduct RIP experiments in accordance with the provided guidelines. Briefly, cells were lysed with lysis buffer. Then, A/G magnetic beads were used for immunoprecipitation of the antibody and the RNA-binding protein. A magnet was used to fix the magnetic beads in place, while the excess material was removed through washing. The RNA was subsequently collected and quantified by qRT-PCR. Both input control and standard IgG antibody control were examined to verify the precision of the signals detected from the RNA bound to the protein.

### Dual-luciferase reporter assays

In the dual-luciferase reporter assays, both wild-type and mutant reporter vectors were constructed by Genecreate (Wuhan, China), with the carrier being pmirGLO. Plates (96-well) were used to seed HEK-293T cells, which were then incubated overnight and transfected with luciferase reporter vectors using Lipo3000 (Invitrogen, L3000–015). The relative luciferase activities were assessed using a Dual-Luciferase Reporter Assay Kit (Promega, E1910) on a Varioskan LUX machine (Thermo Fisher Scientific) after 48 h of incubation. Firefly luciferase activity was normalized to that of Renilla (pRL-TK) luciferase, and fold-changes in luciferase values were calculated.

### Mouse experiments

Animal experiments were conducted with the approval of the Sun Yat-sen University Institutional Animal Care and Use Committee (Approval Number: SYSU-IACUC-2023-001764, 22080B). Four-week-old BALB/c mice and BALB/c-nude mice were purchased from GemPharmatech (China). BALB/c mice and BALB/c-nu mice were subcutaneously injected with Renca cells stably expressing *circGramd4* (5 × 10^5^ cells/100 μL). Tumor volume was measured every 3 days starting from the 7th day after injection (tumor volume = 1/2 × length × width^2). After 21 days, the mice were euthanized, and subcutaneous tumors were dissected to measure tumor weight. Flow cytometry was used to assess the functionality of CD8^+^ T cells infiltrating the tumor. For the construction of PDX models, NCG mice were also purchased from GemPharmatech. Fresh human RCC tumor tissue fragments were subcutaneously transplanted into NCG mice. When the transplanted tumor reached a size of 100 mm^3^, the NCG mice were euthanized, and the transplanted tumors were isolated. The tumors were then re-cut into 1-mm^3^ fragments and implanted into the next generation of NCG mice. After several generations of effort, the PDX model was successfully established. Once the subcutaneous tumor volume reached 100 mm^3^, the mice were randomly divided into four treatment groups: IC+si-nc+IgG, IC+*si-circGRAMD4*+IgG, IC+*si-*nc+anti-PDCD1, and IC+si*-circGRAMD4*+anti-PDCD1. To reconstitute the immune system in NCG mice, human dendritic cells (approximately 0.5 × 10^6^ cells/mouse) and tumor-specific CD8^+^ T cells (approximately 2.5 × 10^6^ cells/mouse) were injected into the mice via the tail vein. Subsequently, subcutaneous tumors were injected with si-nc or *si-circGRAMD4* (5 nmol/mouse, RiboBio, China) every 4 days for a total of 6 injections, and intraperitoneal injections of IgG isotype control (100 μg/mouse, BioXcell, BE0083) or anti-human PDCD1/PD-1 antibodies (100 μg/mouse; BioXcell, BE0188) were performed every 4 days for a total of 7 injections. Tumor volume was measured weekly, and after 4 weeks, the mice were euthanized, and subcutaneous tumors were dissected and weighed. Flow cytometry was used to assess the functionality of CD8^+^ T cells infiltrating the tumor, and immunohistochemistry was performed to evaluate protein expression levels in the tumor tissue.

### Immunohistochemistry (IHC)

First, the paraffin embedded tissue was dewaxed by baking and soaking in xylene. The tissues were then dehydrated with gradient alcohol, followed by antigen repair and endogenous peroxidase blockade. Next, incubation overnight using primary antibodies targeting MKI67/Ki67 (CST, 9129S), LC3B (CST 83,506), MHC-I (abcam, ab134189), NBR1 (Proteintech 16,004–1-AP) and RBM4 (Proteintech 11,614–1-AP) antibodies at 4°C overnight, and apply secondary antibodies the next day, followed by DAB staining, hematoxylin staining of cell nuclei, dehydration and sealing after differentiation. Pictures were gathered utilizing a completely automated digital scanner for pathological sections (Kfbio, KF-PRO-020), and the intensity of IHC staining was evaluated.

### Statistical analysis

Statistical analysis was performed using SPSS 25.0 software. The data were presented as mean ± standard deviation (SD). In vitro experiments were conducted with a minimum of three independent biological replicates. To compare two groups, we employed two independent sample t-tests to assess statistical significance. For multi-group comparisons, analysis of variance (ANOVA) was used. Survival analysis was conducted using Kaplan-Meier survival curves with log-rank tests. Correlation analysis was performed using Pearson correlation for continuous variables and Spearman correlation for discontinuous variables. A P-value less than 0.05 was considered statistically significant, statistical significance was shown as *(*p* < 0.05), ** (*p* < 0.01) or *(*p* < 0.001).

## Supplementary Material

supplementary materials R3.docx

## Data Availability

All data needed to evaluate the conclusions in the paper are present in the paper and/or the Supplementary Materials. Additional data related to this paper may be requested from the authors.
