# Supplementary material for: CircRNA GRAMD4 induces NBR1 expression to promote autophagy and immune escape in renal cell carcinoma
Source: Autophagy. 2025 May 15;21(11):2332–52. doi: 10.1080/15548627.2025.2503560 (PMC12674463; doi:10.1080/15548627.2025.2503560)
Supplement: supplementary materials R3.docx [file KAUP_A_2503560_SM1512.docx]

**Supplementary Materials for**

**CircRNA *GRAMD4* induces *NBR1* expression to promote autophagy and immune escape in renal cell carcinoma**

**Authors:** Mi Zhou^a,1^, Minyu Chen^b,1^, Zhousan Zheng ^a, 1^, Qihao Li^b,1^, Lican Liao ^a^, Yunfei Wang ^a^, Yi Xu ^c^, Guannan Shu^d^, Junhang Luo ^b,*^, Taowei Yang^b,*^, Jiaxing Zhang^a,*^

^*^Corresponding author: Junhang Luo, Taowei Yang, Jiaxing Zhang

**Table S1.** Primers used in this study.

| Primers | Sequence (5′-3′) |
| --- | --- |
| *Hsa_circ_0001250* F | TGGAAAAACATTTCTTACGGAACCC |
| *Hsa_circ_0001250* R | GGGATTTCGTCGCTGCATTC |
| *GRAMD4* F | TCTCTGCCTTATCCGACTGGT |
| *GRAMD4* R | GGCACGATGCTCCACTGTAT |
| Human *GAPDH* F | TGTGGGCATCAATGGATTTGG |
| Human *GAPDH* R | ACACCATGTATTCCGGGTCAAT |
| *Mmu_circ_0005931* F | AAAGCACTTTCTTCGGAGCC |
| *Mmu_circ_0005931* R | GGATCTCGTCTCCGCACTC |
| *Gapdh* F | AGGTCGGTGTGAACGGATTTG |
| *Gapdh* R | GGGGTCGTTGATGGCAACA |
| *RBM4* F | AGAGTGTCCGATAGATCGTTCA |
| *RBM4* R | GAATCCCCATAGCTCATGGTG |
| *NBR1* F | AGATGGCAGTTAAACAGGGAAAC |
| *NBR1* R | GTGGGGCTTCATCAACGACA |

**Table S2.** Correlations between *circGRAMD4* expressions and clinical characteristics of RCC patients in the SYSU Cohort.

| **Parameters** | **Number**  **of cases** | ***CircGRAMD4*** | | ***P* Value** |
| --- | --- | --- | --- | --- |
|  |  | **Low** | **High** |  |
| Age (year) |  |  |  | 0.837 |
| <60 | 65 | 33 | 32 |  |
| ≥60 | 37 | 18 | 19 |  |
| Gender |  |  |  | 0.262 |
| Female | 27 | 16 | 11 |  |
| Male | 75 | 35 | 40 |  |
| TNM stage |  |  |  | <0.001 |
| I | 74 | 45 | 29 |  |
| II+III | 28 | 6 | 22 |  |


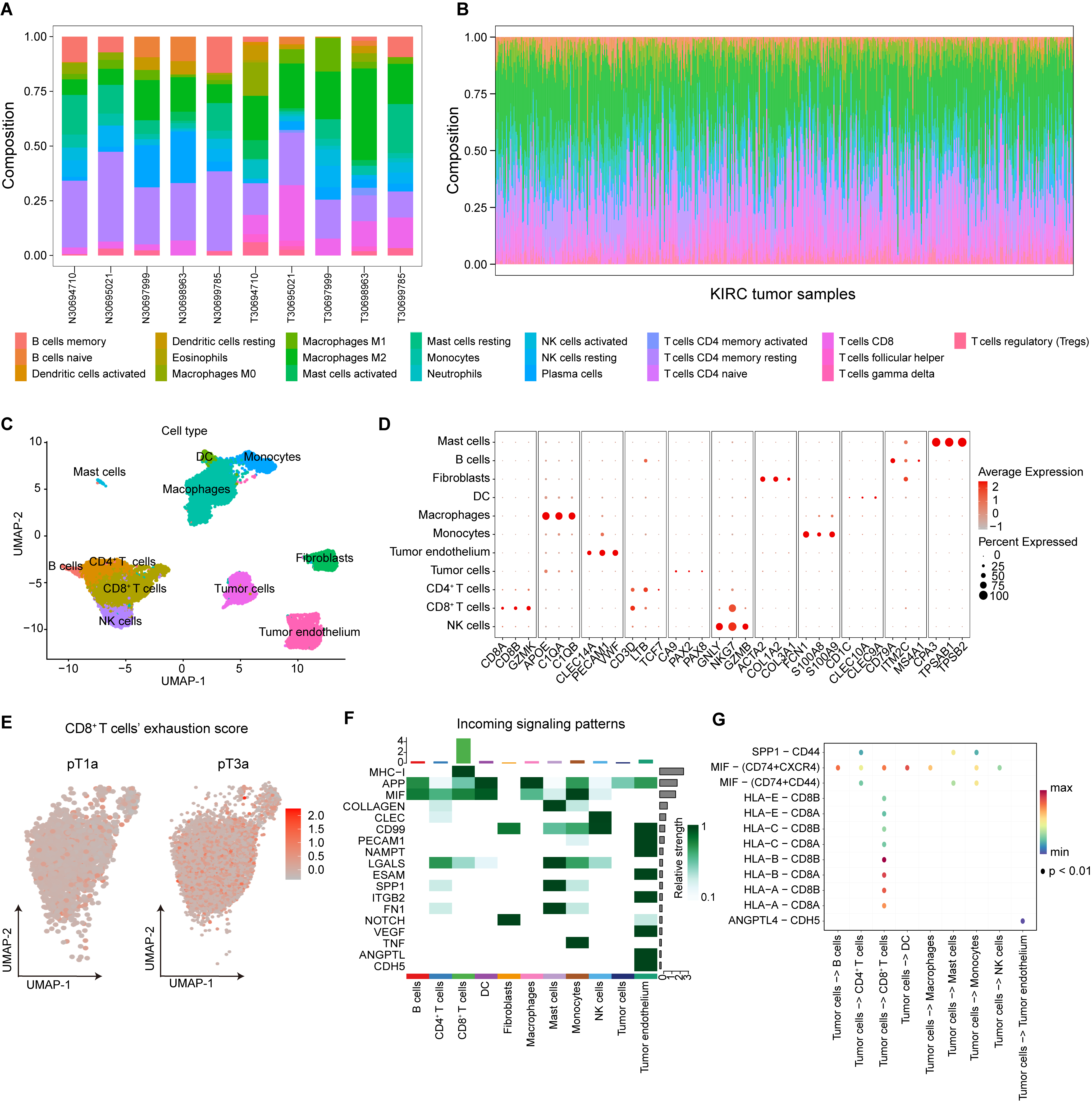


**Figure S1.** Analysis of immune cell infiltration, CD8^+^ T cell exhaustion, and intercellular communication in the RCC TME. (**A**) The CIBERSORT algorithm analysis showed the proportion of immune cells in 5 pairs of RCC. (**B**) The CIBERSORT algorithm analysis showed the proportion of immune cells in 542 KIRC datasets from TCGA. (**C**) UMAP showing different cell populations which have been identified. (**D**) Dot plots showing markers of different cell populations. (**E**) Enrichment pattern of Exhaustion score in the UMAP plot. (**F**) Heatmap showing the incoming signaling patterns in RCC tumor tissues at pT3a stage. (**G**) The bubble chart illustrates the ligands and receptors involved in the communication between tumor cells and other cells within the TME.


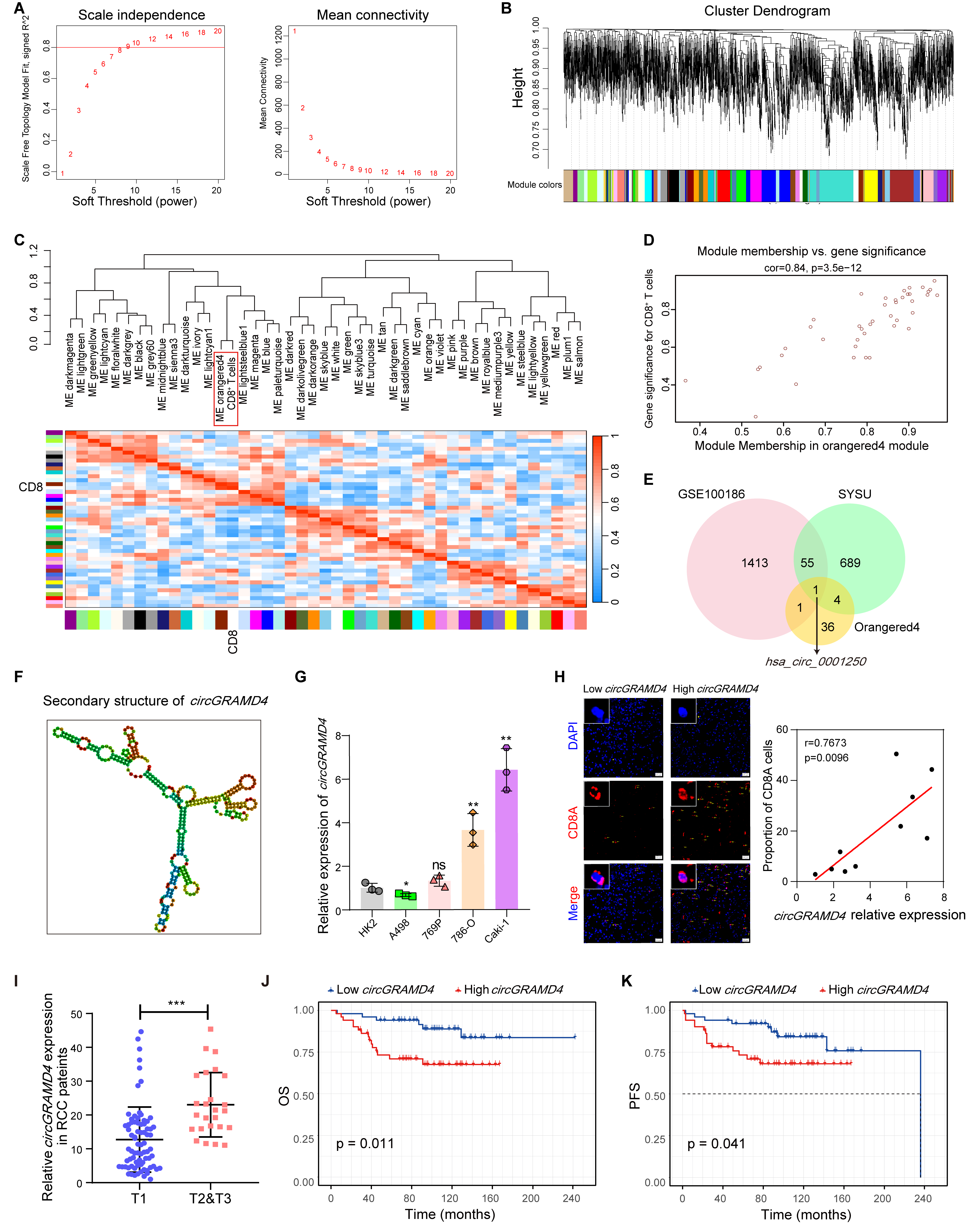


**Figure S2.** *CircGRAMD4* has been identified as a circRNA associated with higher CD8^+^ T cell infiltration and poorer patient prognosis. (**A**) Scale independence and Mean connectivity of circRNA sequencing data from five RCC tissues. (**B**) Cluster dendrogram for circRNA module recognition. (**C**) Dendrogram and correlation heat map of CD8^+^ T cell content and circRNA module. (**D**) 42 hub circRNAs in orangered4 module (gene significance > 0.91, module membership correlation=0.84 and q weighted < 0.01). (**E**) Venn plot of circRNA in orangered4 module and upregulated circRNAs (log2 fold change > 1, and p < 0.05) in our sequencing results and GSE100186 dataset. (**F**) Schematic diagram of the secondary structure of *circGRAMD4*. (**G**)The expression level of *circGRAMD4* in normal kidney cell line HK2 and different RCC cell lines, measured by qRT-PCR. (**H**) IF analysis showed a positive correlation between the expression level of *circGRAMD4* and the proportion of CD8^+^ T cell infiltration. Scale bar: 20 μm. (**I**) Comparison of differences in *circGRAMD4* expression levels within RCC tissues between T1 and T2&T3. Kaplan–Meier analyses of OS (**J**) and PFS (**K**) in high and low *circGRAMD4* expression groups according to median *circGRAMD4* expression in tumors. Data are shown as mean± SD; *p < 0.05, **p < 0.01, ***p < 0.001.


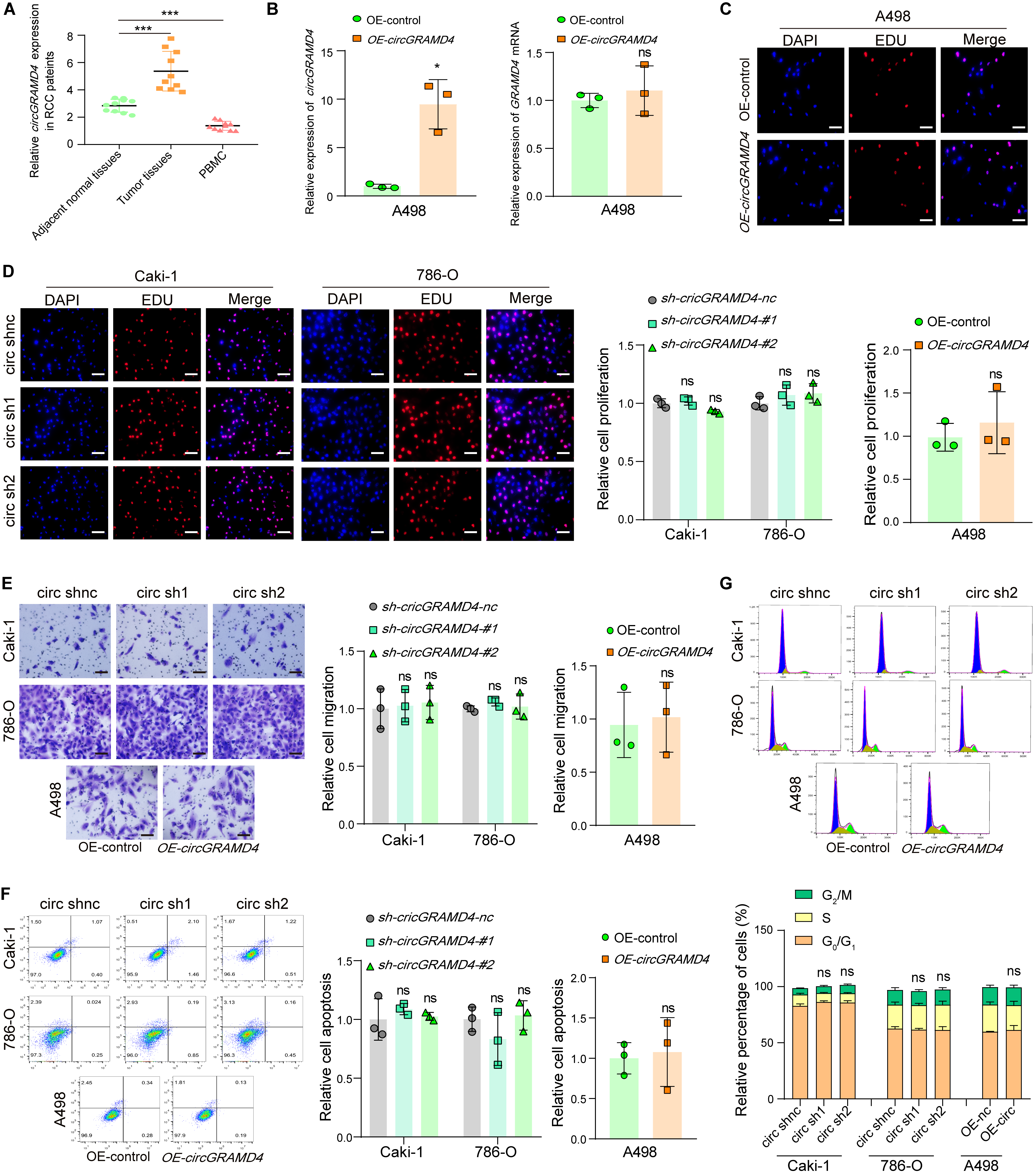


**Figure S3.** In vitro RCC cells, altering the expression of *circGRAMD4* did not significantly impact cell proliferation, migration, apoptosis, or cell cycle. (**A**) qRT-PCR experiment detected the expression levels of *circGRAMD4* in the tumor tissues, adjacent normal tissues and PBMC of RCC patients. (**B**) *CircGRAMD4* overexpression efficiency was measured by qRT-PCR in A498 cell line. (**C**) and (**D**) EDU assay showed that knockdown or overexpression of *circGRAMD4* in vitro RCC cells had no significant effects on cell proliferation. (**E**) Transwell experiment showed that knockdown or overexpression of *circGRAMD4* in vitro RCC cells had no significant effects on cell migration. (**F**) and (**G**) Flow cytometry analysis showed knockdown or overexpression of *circGRAMD4* in vitro RCC cells had no significant effects on cell apoptosis and cell cycle. Data are shown as mean± SD; *p < 0.05, **p < 0.01, ***p < 0.001.


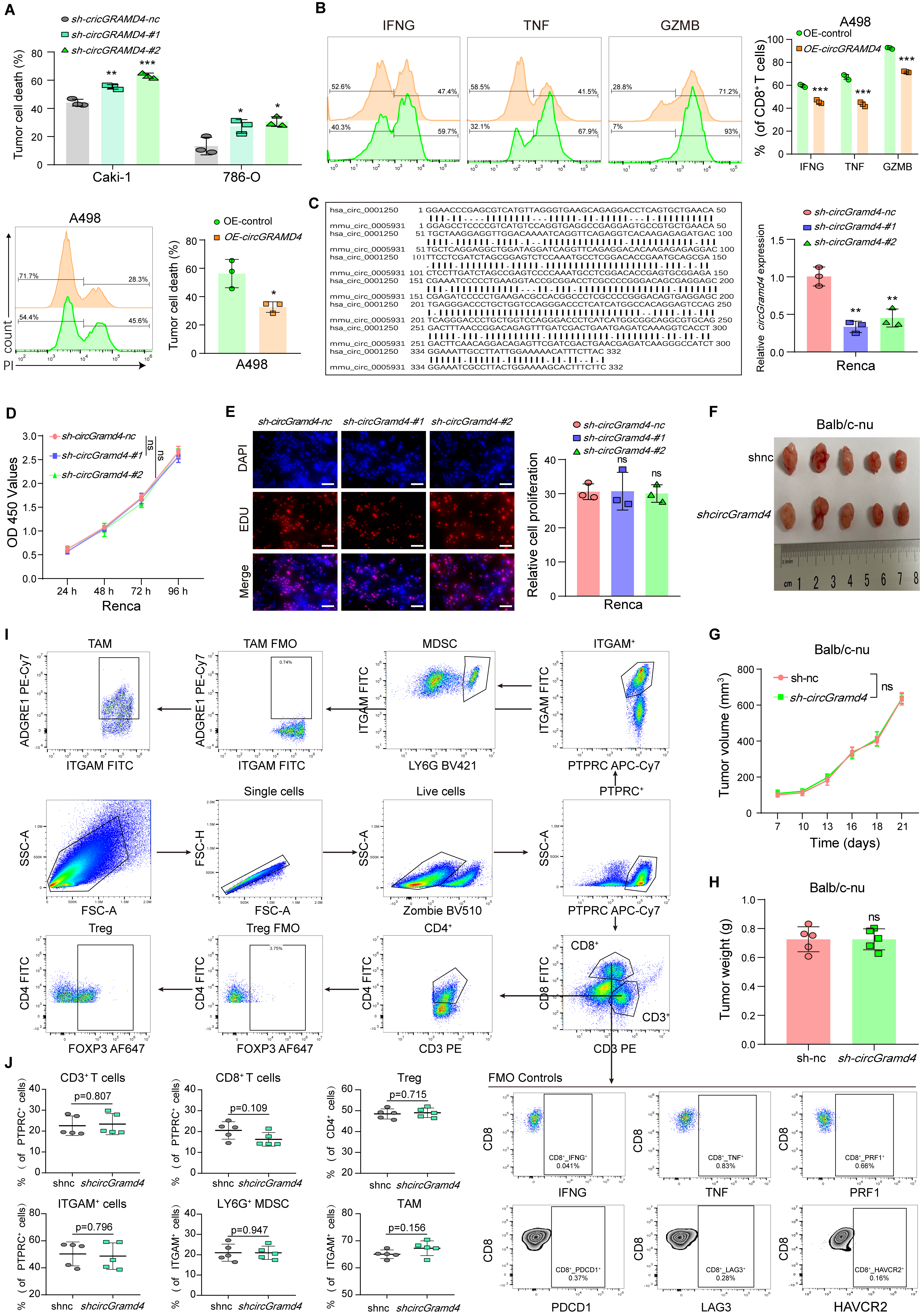


**Figure S4.** The expression level of *circGRAMD4* in renal cancer cells can affect the function of CD8^+^ T cells. (**A**) Representative flow cytometry images of death rate (PI^+^) of primary kidney tumor cells with or without *circGRAMD4* knockdown/overexpression cocultured with primary kidney tumor-specific CD8^+^ T cells. (**B**) Representative images and statistical quantification of the FACS analysis of the percentage of IFNG^+^, TNF^+^, and GZMB^+^ CD8^+^ T cells cocultured with A498 cells with or without *circGRAMD4* overexpression. (**C**) Left：*hsa_circ_0001250* (*circGRAMD4*) and *mmu_circ_0005931* (*circGramd4*) gene sequences. Right: The *circGramd4* knockdown efficiency was measured by qRT-PCR in Renca cell line. (**D**) CCK8 and (**E**) EDU assays showed Renca cell proliferation after transfection with shnc or sh*circGramd4*. (**F**) Images of the collected subcutaneous xenograft tumors from Balb/c-nu mice. (**G**) Record the tumor volume of tumor bearing Balb/c-nu mice every 3 days. (**H**) Eventual weights of subcutaneous xenograft tumors. (**I**) Gating strategy used to define lymphoid subsets in the Balb/c Subcutaneous tumor model. The FMO control represents a fully stained sample minus the indicated antibody. (**J**) Statistical results of the proportion of tumor infiltrating lymphocytes in subcutaneous tumors. Treg: T regulatory cells; MDSC: myeloid-derived suppressor cells; TAM: tumor-associated macrophages. Data are shown as mean± SD; *p < 0.05, **p < 0.01, ***p < 0.001.


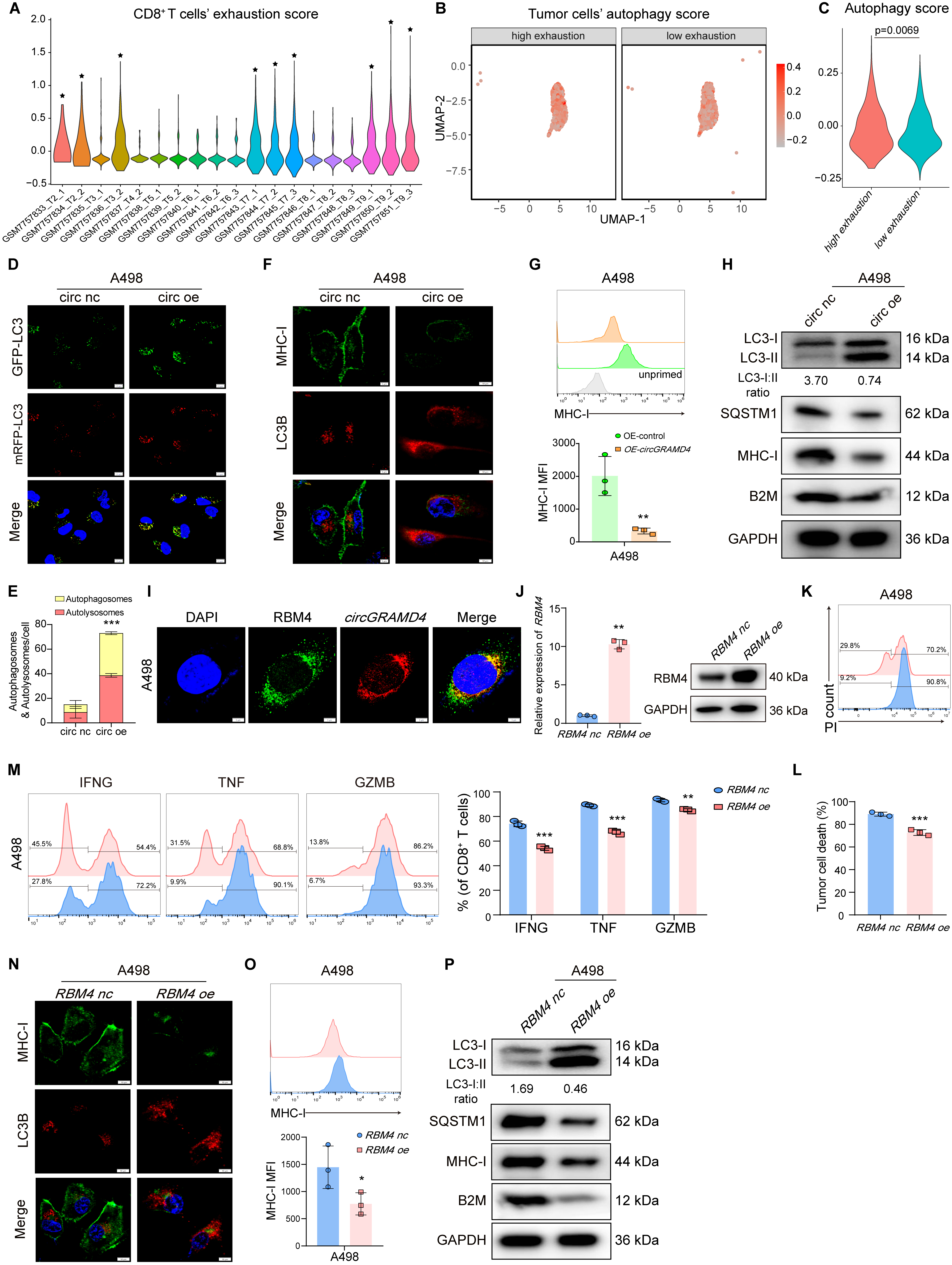


**Figure S5.** *CircGRAMD4* and RBM4 synergistically regulate the expression of MHC-I in RCC and affect CD8^+^ T cell function. (**A**) Violin plot of CD8^+^ T cell exhaustion score of each sample in GSE24299 dataset. ★ indicates the group with high exhaustion score. (**B**) UMAP diagram shows the enrichment pattern of tumor cell autophagy score in CD8^+^ T cell high exhaustion samples and low exhaustion sample. (**C**) Violin plot of tumor cell autophagy scores in CD8^+^ T cell exhaustion_high and exhaustion_low group. (**D**) and (**E**)GFP-mRFP-LC3 labeled A498 cells were infected with circnc or *circGRAMD4*-oe plasmid. Autophagic flux was analyzed by confocal microscopy. Scale bar: 5 μm. (**F**) Detection of MHC-I and LC3B levels in A498 cells using immunofluorescence assay. Scale bar: 5 μm. (**G**) Representative images and statistical quantification of the FACS analysis of MHC-I molecules on cell surface. (**H**) LC3-I:II, SQSTM1, MHC-I, B2M and GAPDH protein expression was measured by western blot. (**I**) IF-FISH assays showed that *circGRAMD4* and RBM4 colocalized in the A498 cells’ cytoplasm. (**J**) RBM4 overexpression efficiency was measured by qRT-PCR and western blot in A498 cell line. (**K**) and (**L**) Representative flow cytometry images of death rate (PI^+^) of A498 cells with or without RBM4 overexpression cocultured with CD8^+^ T cells. (**M**) Representative images and statistical quantification of the FACS analysis of the percentage of IFNG^+^, TNF^+^, and GZMB^+^ CD8^+^ T cells cocultured with A498 cells with or without RBM4 overexpression. (**N**) Detection of MHC-I and LC3B levels in A498 cells infected with *RBM4-nc* or *RBM4-oe* plasmid using immunofluorescence assay. Scale bar: 5 μm. (**O**) Representative images and statistical quantification of the FACS analysis of MHC-I molecules on cell surface. (**P**) LC3-I:II, SQSTM1, MHC-I，B2M and GAPDH protein expression was measured by western blot. Data are shown as mean± SD; *p < 0.05, **p < 0.01, ***p < 0.001.


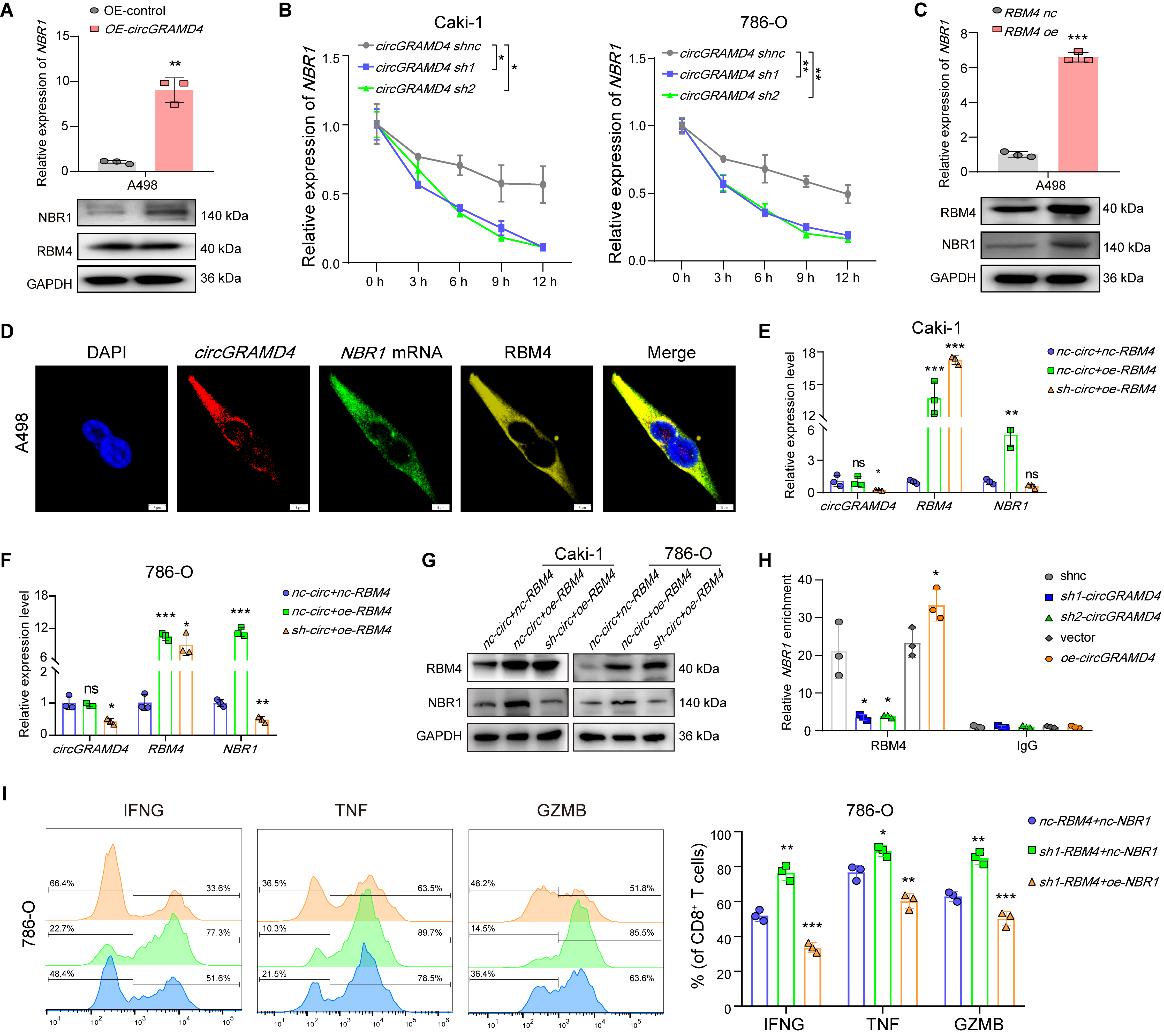


**Figure S6.** *CircGRAMD4* and RBM4 indirectly affect the anti-tumor effect of CD8^+^ T cells by affecting the expression of *NBR1*. (**A**) *NBR1* mRNA levels and protein levels were measured after *circGRAMD4* overexpression. (**B**) Reduced stability of *NBR1* mRNA upon *circGRAMD4* knockdown**.** (**C**) *NBR1* mRNA levels and protein levels were measured after RBM4 overexpression. (**D**) IF-FISH assays showed that *circGRAMD4*, *NBR1* mRNA and RBM4 protein colocalized in the cytoplasm. (**E**) and (**F**) qRT-PCR detected the relative expression level of *circGRAMD4*, *RBM4* and *NBR1* after cells were transfected with control vector, oe-*RBM4* alone or oe-*RBM4* plus sh-*circGRAMD4*. (**G**)Western blot detected the protein level of RBM4 and NBR1 after cells were transfected with control vector, oe-*RBM4* alone or oe-*RBM4* plus sh-*circGRAMD4*. (**H**) RIP assays showing the association of *RBM4* with *NBR1* upon *circGRAMD4* silencing or overexpression. (**I**) Representative images and statistical quantification of the FACS analysis of the percentage of IFNG^+^, TNF^+^, and GZMB^+^ CD8^+^ T cells cocultured with 786-O cells with *sh-RBM4* and/or *NBR1* overexpression. Data are shown as mean± SD; *p < 0.05, **p < 0.01, ***p < 0.001.


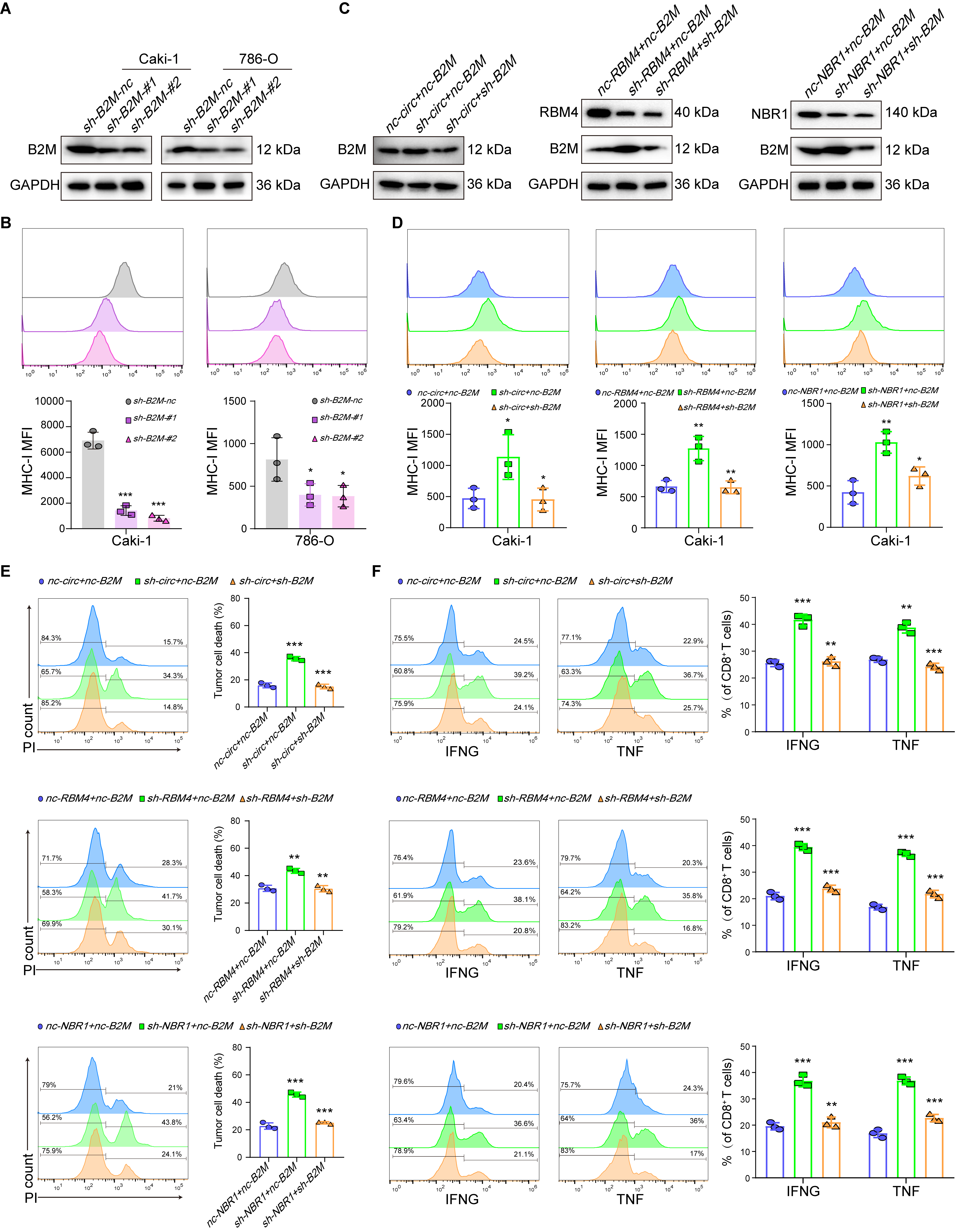


**Figure S7.** Revealing through B2M knockout rescue experiments that the *circGRAMD4-*RBM4*-NBR1* axis mediates tumor cell immune escape by regulating MHC-I levels. (**A**) Western blot results show the B2M knockdown effect. (**B**) Representative images and statistical quantification of the FACS analysis of the MHC-I molecules on cell surface after B2M knockdown. (**C**) Western blot results show the RBM4, NBR1 and B2M knockdown effect. (**D**) Representative images and statistical quantification of the FACS analysis of the MHC-I molecules on cell surface after performing rescue experiments. (**E**) Representative flow cytometry images of death rate (PI^+^) of Caki-1 cells cocultured with CD8^+^ T cells. (**F**) Representative images and statistical quantification of the FACS analysis of the percentage of IFNG^+^ and TNF^+^ CD8^+^ T cells cocultured with Caki-1 cells. Data are shown as mean± SD; *p < 0.05, **p < 0.01, ***p < 0.001.

**Supplemental methods**

***Assay for 5-Ethynyl-2′-Deoxyuridine (EdU)***

Inoculate the cells in a confocal dish (NEST, 801001-1-N2) and wait for the cells to adhere to the bottom. Labeling cells with EdU (Beyotime, C0078S) for 2 h, then fixed cells for 15 min, and permeate cells using 0.3% Triton X-100. Following a rinse with PBS containing 3% BSA (ABCONE, B24726-100G), a 0.5 mL click additive solution was added to each well and incubated at room temperature 30 min in the dark. The cells were then counterstained with 1× Hoechst 33342 for 10 min.

***CCK-8 Assay***

Inoculate cells (5000/well) into a 96-well plate, with 6 wells per group. Timing was started from after cell apposition, we added CCK-8 reagent (MCE, HY-K0301) to each well every 0, 24, 48, 72, and 96 h, and then incubated with cells at 37°C for 2 h. Finally, microplate reader (V arioskan LUX, Thermo Scientific, USA) detected the absorbance of cells at 450 nm.

***Transwell assay***

Add complete culture medium (600-800 ul/well) to a 24-well plate, then gently place the transwell chamber in the 24-well plate. Digest the cells to be tested with trypsin and resuspend them in serum-free culture medium. Count the cells and adjust the concentration of cells in each group to about 500,000 cells/mL. Take 100-150 uL of the cell suspension and drop it into the center of the transwell chamber. Cultivate in a cell culture incubator for 12-24 h. After removing the chamber, fix the cells with 4% paraformaldehyde, gently wipe off the cells inside the chamber with a cotton swab, and finally stain the cells with crystal violet.
